# Evaluation of Item Fit With Output From the EM Algorithm: RMSD Index Based on Posterior Expectations

**DOI:** 10.1177/00131644251369532

**Published:** 2025-10-04

**Authors:** Yun-Kyung Kim, Li Cai, YoungKoung Kim

**Affiliations:** 1 University of California, Los Angeles, CA, USA; 2 College Board, New York, NY, USA

**Keywords:** item fit, posterior predictive model checking (PPMC), EM algorithm, root mean squared deviation (RMSD), receiver operating characteristic (ROC) curve analysis, response surface analysis

## Abstract

In item response theory modeling, item fit analysis using posterior expectations, otherwise known as pseudocounts, has many advantages. They are readily obtained from the E-step output of the Bock–Aitkin Expectation-Maximization (EM) algorithm and continue to function as a basis of evaluating model fit, even when missing data are present. This paper aimed to improve the interpretability of the root mean squared deviation (RMSD) index based on posterior expectations. In Study 1, we assessed its performance using two approaches. First, we employed the poor person’s posterior predictive model checking (PP-PPMC) to compute their significance levels. The resulting Type I error was generally controlled below the nominal level, but power noticeably declined with smaller sample sizes and shorter test lengths. Second, we used receiver operating characteristic (ROC) curve analysis (±) to empirically determine the reference values (cutoff thresholds) that achieve an optimal balance between false-positive and true-positive rates. Importantly, we identified optimal reference values for each combination of sample size and test length in the simulation conditions. The cutoff threshold approach outperformed the PP-PPMC approach with greater gains in true-positive rates than losses from the inflated false-positive rates. In Study 2, we extended the cutoff threshold approach to conditions with larger sample sizes and longer test lengths. Moreover, we evaluated the performance of the optimized cutoff thresholds under varying levels of data missingness. Finally, we employed response surface analysis (±) to develop a prediction model that generalizes the way the reference values vary with sample size and test length. Overall, this study demonstrates the application of the PP-PPMC for item fit diagnostics and implements a practical frequentist approach to empirically derive reference values. Using our prediction model, practitioners can compute the reference values of RMSD that are tailored to their dataset’s sample size and test length.

## Introduction

Given the widespread use of item response theory (IRT) models, evaluation of IRT model fit is pivotal to any IRT-based measurement activities. Especially, an item-level evaluation of fit is of great interest to practitioners who desire to identify items that do not sufficiently conform to the fitted model. Evaluation of an item fit, however, is not as straightforward as evaluating an overall model fit. It requires that examinees’ categorical responses to the item (e.g., correct or incorrect, in case of a dichotomously scored item) be rearranged in a way that they can be compared with a model prediction that is represented on a continuous scale of a latent variable θ that underlies the item responses.

Various item fit evaluation methods have been proposed to address the challenge, among which chi-square type indices are the most common. The indices primarily differ in the ways they sort examinees into subgroups. Examinees were sorted into a fixed or varying number of intervals based on their θ estimates (Bock, 1972; Yen, 1981) or were grouped based on their summed scores or number-correct scores (Kang & Chen, 2008; Orlando & Thissen, 2000, 2003). The summed score approach is advantageous in that the sorting no longer depends on the choice of models and intervals, but it is prone to misclassification of examinees, especially when summed scores are imprecise approximations of latent variable scores due to short test lengths or high degree of data missingness.

An alternative approach is to use posterior expectations, also known as pseudocounts or pseudo-observed frequencies (Donoghue & McClellan, 1999, 2003; Li, 2005; Stone, 2000, 2003). The posterior expectations are represented on quadrature points, a set of discrete values that approximate the continuous scale of θ to a practical degree of accuracy. They are an accumulation of the posterior probabilities of each examinee’s latent variable score across the quadrature points, given the prior distribution, observed item responses, and model parameter estimates. Since each examinee contributes probabilistically across all quadrature points—rather than being assigned to a single point—the resulting aggregate distribution forms a smooth approximation of the latent trait continuum. This way, posterior expectations enable an optimal and continuous reallocation of examinees across the θ continuum without relying on arbitrary subgroup classifications. This smoothing is especially beneficial under small sample size or severe missingness due to test design (e.g., van der Linden et al., 2004). Moreover, posterior expectations reflect the uncertainties in the score estimates, which make them the source for point estimates of θ , such as expected a posteriori and maximum a posteriori scores, as well as associated standard errors. Finally, they are natural by-products of the Bock–Aitkin Expectation-Maximization (EM) algorithm (Bock & Aitkin, 1981), most widely used for IRT estimation, thereby requiring no additional computation.

Given rich information and the ease of access, posterior expectations have been widely used to visualize observed probabilities alongside model-implied probabilities (e.g., Allen et al., 1999; Eckerly et al., 2022; van Rijn et al., 2016). This approach is also described as comparing the empirical or observed item response functions (IRFs) or tracelines with their theoretical or expected counterparts. The agreement or disagreement between the two curves is typically assessed through a visual inspection or “eyeballing,” due to the challenge in performing a statistical test for misfit.

The most prominent test quantity that can support—if not replace—the visual inspection is the root mean squared deviation (RMSD) index, which has gained increasing attention in recent years (Köhler et al., 2020; Robitzsch, 2022). The RMSD quantifies the discrepancy between the expected and observed IRFs, weighted by the population density. That is, item fit is assessed by how much the observed IRF deviates from the expected IRF, with greater weight given to regions where the population density is higher. In this way, the RMSD effectively captures the data-model fit in a relevant manner. Note that the RMSD has also appeared in literature as the root integrated squared error (RISE) index that quantifies the discrepancy between parametric and nonparametric IRFs (e.g., Douglas & Cohen, 2001; Lee et al., 2009). The RMSD can be understood as a special case of RISE where the nonparametric IRF is estimated using posterior expectations (e.g., Sueiro & Abad, 2011). In this study, we focus on the RMSD as it does not require additional fitting of the nonparametric IRF.

Despite the ease of computation and the meaningful interpretation offered by the RMSD, its null distribution remains unknown due to the impact of sampling error, estimation error, and the dependencies among the pseudocounts (attributable to each examinee probabilistically contributing to all quadrature points). Moreover, the RMSD is not interpretable as an effect size for misfit (i.e., a consistent measure that informs the magnitude of misfit; Köhler et al., 2021) because its size depends on the characteristics of the data, such as sample size and test length (Köhler et al., 2020;, 2021; Robitzsch, 2022). To address the challenge, a parametric bootstrap method was used to compute significance values for the RMSD (Köhler et al., 2020;, 2021; Sueiro & Abad, 2011), which demonstrated unsatisfactory power under small to moderate sample sizes and shorter test lengths. Other efforts have aimed to analytically derive the asymptotic null distribution (Donoghue & McClellan, 1999, 2003), but the required task of computing the covariance matrix of pseudocounts is computationally intensive and thus has not been widely implemented in practice. Even after the computational burden was alleviated by replacing the full covariance matrix with its observed counterpart (Li, 2005), the approximation was prone to introducing additional sources of error. More fundamentally, the existing methods for computing the significance values rely on the assumption that item parameters are fixed and known, which is highly restrictive in applied settings.

In this study, we demonstrate two approaches to address the limitation. First, we demonstrate the implementation of the poor person’s posterior predictive model checking (PP-PPMC) introduced by Lee et al. (2016) to compute significance values for the RMSD. The PP-PPMC is a frequentist simplification of posterior predictive model checking (PPMC; Gelman et al., 1996; Rubin, 1984), a prominent Bayesian model diagnostic tool. PPMC has a strong theoretical basis and provides graphical and numerical evidence about model fit, while organically accounting for sampling and estimation errors. That is, it no longer relies on the unrealistic assumption that item parameters are fixed and known. Despite the merits, since PPMC requires simulated draws from a posterior predictive distribution, it has almost exclusively been applied to Bayesian IRT contexts (e.g., Joo & Lee, 2022; Levy et al., 2009; Sinharay, 2005, 2006; Sinharay et al., 2006). The PP-PPMC (Lee et al., 2016), however, enabled the PPMC procedure in models estimated using maximum likelihood (ML). It was shown that Bayesian estimation can be bypassed by approximating the posterior distribution with a multivariate normal distribution, using ML estimates as the mean and the corresponding variance–covariance matrix as the covariance. While the original development took place in the context of structural equation modeling, Kuhfeld (2019) applied the PP-PPMC to the context of IRT estimated using full-information ML to test for local independence, where it was once again confirmed that the multivariate normal approximation of the posterior distribution performed well even with small sample sizes. In this study, we extended the application of the PP-PPMC to item fit evaluation using the RMSD. This application enabled us to assess the performance of the RMSD by examining the distribution of its significance levels under null conditions.

Second, we derived reference values (cutoff thresholds) for the RMSD to facilitate its use. We utilized the item fit values generated via simulation study to construct an empirical distribution of the RMSD under null and non-null conditions. Then, we identified the optimal reference values using receiver operating characteristic (ROC) curve analysis (Metz, 1978) and fitted a model by response surface analysis (Box, 1954) to generalize the optimized reference values to conditions beyond those in the simulation study. This procedure verified the performance of the RMSD and made available the reference values that practitioners can readily adopt to interpret their RMSD values. In addition, the performance of the optimized reference values was evaluated in the presence of missing data commonly found in large-scale assessments.

The rest of the paper is structured as follows. We first describe the computation of the RMSD and outline the two approaches in detail, along with their guiding philosophy. Next, we present two simulation studies that implement the methods. In Study 1, we compared the performance of the two approaches: the PP-PPMC method and the application of optimized reference values. In Study 2, we derived the optimized reference values for conditions not covered in Study 1, with greater emphasis on large-scale assessment contexts characterized by larger sample sizes, longer test lengths, and higher levels of data missingness. The simulation studies are followed by an empirical example that illustrates the application of the findings. We conclude with summary and discussions.

## Computation of Pseudocounts and RMSD

Suppose there are dichotomously scored items ( i=1,…,n ), persons or examinees ( j=1,…,N ), and a unidimensional IRT model with m parameters. The distribution of a latent variable θ is approximated by a set of quadrature points $X_{q}$ for q=1,…,Q . Let $p_{\mathrm{iq}}$ be an observed probability of endorsing an item i at $\theta =X_{q}$ , and $\pi_{\mathrm{iq}}$ be an expected or model-predicted probability of endorsing an item i at $\theta =X_{q}$ . The comparison of the observed and expected probabilities on the lowest response category (e.g., incorrect response in dichotomously scored items) is omitted to prevent redundancy.

### Pseudocounts

Donoghue and McClellan (1999, 2003) characterized pseudocounts as compilations across *response patterns* to items except for the target item. Under the framework, a pseudocount for item i at quadrature point q is $s_{\mathrm{iq}}=\sum_{t=1}^{T}n_{t}p_{\mathrm{itq}}$ , where $n_{t}$ is the number of examinees with a response pattern $y_{t}$ on remaining items (i.e., all items except for item i ),

(1) $$p_{\mathrm{itq}}=P\left(\theta =X_{q}|U_{i}=1,y=y_{t}\right),$$

is a posterior probability of $\theta =X_{q}$ given a correct response to item i and response pattern of $y_{t}$ to the remaining items, and T is the total number of unique response patterns. In a matrix form, a pseudocount vector $s_{i}$ can be written as

(2) $$s_{i}=P_{i}^{'}n={\left(\begin{array}{ccc} p_{i11} & \cdots & p_{i1Q}\\ \vdots & \ddots & \vdots \\ p_{\mathrm{iT}1} & \cdots & p_{\mathrm{iTQ}} \end{array}\right)}^{'}\left(\begin{array}{c} n_{1}\\ \vdots \\ n_{T} \end{array}\right)$$

where the frequency vector n follows a multinomial distribution. As the number of items increases, the computational burden of carrying the matrix $P_{i}$ of size T×Q becomes very large.

Alternatively, Li (2005) reformulated $s_{i}$ as compilations across *persons* instead of response patterns. Each person’s contribution to pseudocounts is referred to as *unit* pseudocounts. A unit pseudocount of person j for item i at quadrature point q is defined as

(3) $$x_{\mathrm{ijq}}=P(\theta =X_{q}|U_{i}=u_{\mathrm{ij}},y=y_{j}).$$

The unit pseudocounts of each person (i.e., $x_{\mathrm{ij}1},\;\ldots ,\;x_{\mathrm{ijQ}}$ ) are normalized to sum up to one, so that every person has the same overall contribution to the pseudocounts. Examinees who do not have a valid response to item i (i.e., missing) do not contribute to the pseudocounts for the item. Likewise, those who responded incorrectly to item i do not contribute to the pseudocounts pertaining to the correct response category of the item. Hence, provided that $N_{i1}$ denotes the number of examinees who responded correctly to item i , only the unit pseudocounts of $N_{i1}$ examinees are compiled to compute $s_{i}$ . That is,

(4) $$s_{i}=X_{i}^{'}1_{N_{i}}={\left(\begin{array}{ccc} x_{i11} & \cdots & x_{i1Q}\\ \vdots & \ddots & \vdots \\ x_{iN_{i}1} & \cdots & x_{iN_{i1}Q} \end{array}\right)}^{'}\left(\begin{array}{c} 1\\ \vdots \\ 1 \end{array}\right).$$

The reformulation has improved computational efficiency by decreasing the size of the matrix from T×Q of $P_{i}$ (Equation [2]) to $N_{i1}\times Q$ of $X_{i}$ (Equation [4]).

When a model is estimated using the EM algorithm (Bock & Aitkin, 1981), the pseudocount vector $s_{i}$ is part of the E-table, a natural by-product of the E-step. Hence, practitioners do not need to compute $s_{i}$ separately if they can retrieve the E-table from the last iteration^1^. Even when E-tables are not retrieved, the pseudocounts can be readily computed using Equation (4) with minimal computational burden.

### RMSD Index

An item fit can be quantified as the discrepancy between the observed and expected probabilities correct: $e_{i}=p_{i}-\Pi_{i}$ . To be explicit about the sources of variance, it can be written as $e_{i}=p_{i}\left(\hat{\omega };Y\right)-\Pi_{i}({\hat{\omega }}_{i})$ , where ${\hat{\omega }}_{i}={\left({\hat{\omega }}_{i1},\;\ldots ,\;{\hat{\omega }}_{\mathrm{im}}\right)}^{\prime }$ is a vector of length m with item parameter estimates pertaining to item i , ω^=({ω^_1^′,…,ω^_n^′) is a vector of length m×n with all item parameter estimates, and Y is the N×n data matrix containing all item responses. Each element of the observed probability vector $p_{i}=(p_{i1},\ldots ,p_{\mathrm{iq}},\ldots ,p_{\mathrm{iQ}})\prime$ is obtained by dividing the sum of unit pseudocounts across examinees who answered item i correctly (i.e., with total count $N_{i1}$ ) by the sum of unit pseudocounts across all examinees with a valid response to the item (with total count $N_{i}$ , where $N_{i}=N_{i1}+N_{i0}$ ). Note that, at quadrature points where the denominator approaches zero, a small constant (e.g., 0.001) must be added to avoid division by zero and to ensure the identifiability of $p_{\mathrm{iq}}$ . The expected probability vector $\Pi_{i}$ is the item characteristic curve (ICC) or traceline fully determined by the associated item parameter estimates. Then, the RMSD is formulated as $\sqrt{e_{i}^{\prime }We_{i}}$ where W is a weight matrix.

The weight matrix can be an identity matrix $Q^{-1}I_{Q}$ if the intention is to weigh the discrepancies at each quadrature point equally. Alternatively, if one wishes to place greater emphasis on the discrepancies in the tails more than those near the center, a weighting scheme of the Anderson–Darling test (Anderson & Darling, 1952, 1954) can be used. However, caution is warranted when applying such weights, as the sum of pseudocounts is typically smaller in the tails, making the observed probabilities less stable. From a substantive perspective, discrepancies in the tails affect relatively fewer examinees, compared to those near the center. For this reason, the population density has most commonly been used as weights (e.g., Köhler et al., 2020, 2021; Robitzsch, 2022; Sueiro & Abad, 2011), reflecting the prevailing consensus that the discrepancies affecting a larger number of examinees should be assigned greater weights. In this study, we also chose to use the population density as weights, given its widespread use. Under the assumption that population distribution is standard normal, W becomes $\mathrm{diag}\left(\phi_{1},\ldots ,\phi_{q},\ldots ,\phi_{Q}\right)$ , where $\phi_{q}$ denotes the normalized standard normal density function, $\phi_{q}=\phi (X_{q})/\sum_{r=1}^{Q}\phi (X_{r})$ .

## Item Fit Index Development

While the RMSD effectively quantifies the deviations indicative of an item fit or misfit, it lacks interpretability on its own. To make the index meaningful, we require either statistical significance levels (i.e., p -values) or reference values that provide the context for how big or small the observed RMSD values are. To address this, we implemented the following two approaches via simulation studies.

One approach is the use of the PP-PPMC method (Lee et al., 2016) to assess the properties of the RMSD, including the distribution of its significance levels under null conditions, Type I error rate, and power. Here, the focus was to compute the significance level of the index for *each* dataset. The significance levels represent the plausibility of the observed discrepancies (quantified by the RMSD in our application) relative to the discrepancies found in hypothetical replications *after* observing each dataset. Thus, as formally introduced later in this section, the replications ( $Y^{\mathrm{rep}}$ ) are sampled from the *posterior predictive distribution* that conditions on the observed data ( $Y^{\mathrm{obs}}$ ) as well as the hypothesized model ( H ), that is, $p\left(Y^{\mathrm{rep}}|Y^{\mathrm{obs}},H\right)$ .

Another approach is to derive optimal reference values by empirically evaluating the diagnostic performance of all possible thresholds. We utilized the observed discrepancies (i.e., the RMSD values) generated via simulation studies to construct an empirical distribution of the RMSD under null and non-null conditions. Here, hypothetical replications were sampled from the *predictive distribution* that conditions on the model only, *before* observing any data, that is, $p\left(Y^{\mathrm{rep}}|H^{\mathrm{true}}\right)$ where $H^{\mathrm{true}}$ is a data-generating model. Such replications lead to “unconditional frequency checks” (Box, 1980; Rubin, 1984, p. 1167). Based on the empirically constructed distribution of the RMSD values under null and non-null conditions, we identified optimal reference values to maximize true-positive rates while minimizing false-positive rates.

Both of these approaches leverage Monte Carlo frequency simulation to approximate and communicate Bayesian answers, aligning with what has been described as “Bayesianly justifiable and relevant frequency calculations” (Rubin, 1984, p. 1152), an “amalgam of Bayesian and frequentist ideas” (Little, 2006, p. 220), or a “frequentist simplification” (Bayarri & Berger, 2004, p. 71). Moreover, they illustrate how the definition of replications should vary depending on the purpose of model checking (Rubin, 1984). In the PP-PPMC approach, replications were drawn from the posterior predictive distribution that conditions on the model *and* the observed data. This approach assesses the significance level of the RMSD observed in a given data, and the ultimate goal is to validate whether the RMSD formalizes the model-data discrepancy in “relevant ways” (Rubin, 1984, p. 1166). In the cutoff threshold approach, replications were drawn from the predictive distribution that conditions on the model *only*, before any data are observed. This approach facilitates the identification of reference values that distinguish the RMSD values observed in correct model specification from those observed under model misspecification. The following subsections elaborate on these approaches in general terms.

### PPMC and PP-PPMC

Given a hypothesized model H , the PPMC compares the observed data $Y^{\mathrm{obs}}$ with replicated data $Y^{\mathrm{rep}}$ (i.e., data predicted by the model) in terms of a user-defined discrepancy measure T(Y,ω) , determined by data Y and model parameters ω. By Bayes’ rule, the posterior distribution of the parameters ω in model H is

(5) $$p\left(\omega |Y^{\mathrm{obs}},H\right)\propto p\left(Y^{\mathrm{obs}}|\omega ,H\right)p\left(\omega |H\right).$$

The replicated data are generated from the posterior predictive distribution, which is the conditional distribution of replicated data $Y^{\mathrm{rep}}$ given the observed data $Y^{\mathrm{obs}}$ and model H :

(6) $$p\left(Y^{\mathrm{rep}}|Y^{\mathrm{obs}},H\right)=\int_{\omega }p\left(Y^{\mathrm{rep}}|\omega ,H\right)p\left(\omega |Y^{\mathrm{obs}},H\right)d\omega .$$

Notice that there are two components determining the posterior predictive distribution. First, $p\left(Y^{\mathrm{rep}}|\omega ,H\right)$ is the sampling distribution of replicated data given the parameters and the model. Second, $p\left(\omega |Y^{\mathrm{obs}},H\right)$ is the posterior distribution of the parameters estimated from the observed data and the model (Equation [5]). In Equation (6), the uncertainty of model parameters or, equivalently, the estimation error is accounted for by integrating out, or averaging over, the plausible values of the model parameters.

When the posterior distribution of the parameters (Equation [5]) is approximated by a multivariate normal distribution with ML parameter estimates as means and associated variance-covariance matrix as covariances, the procedure is referred to as PP-PPMC (Lee et al., 2016). The approximation relies on the posterior distribution being asymptotically normal, and this strategy can be described as “employing frequentist estimation and treating the answers as Bayesian results” (e.g., Levy & McNeish, 2023). The normal approximation to the posterior distribution has also been used in service of computing Bayes factors (Gu et al., 2019). The accuracy of the normal approximation may be reduced for a small sample size. However, in the context of IRT, Kuhfeld (2019) demonstrated that, even with a sample size as small as 250, the normal approximation to the posterior closely matched the posterior distribution obtained from Bayesian estimation.

To compare observed data with replicated data in terms of the discrepancy measure T(Y,ω) , the following steps are taken. First, a sufficiently large number of plausible parameter vectors $\omega^{l}$ for l=1,…,L are drawn from $p\left(\omega |Y^{\mathrm{obs}},H\right)$ . Second, for each plausible parameter vector $\omega^{l}$ , replicated data $Y^{\mathrm{rep},l}$ are drawn from $p\left(Y^{\mathrm{rep},l}|\omega^{l},H\right)$ . Third, for each plausible parameter vector $\omega^{l}$ , the realized test quantity $T\left(Y^{\mathrm{obs}},\omega^{l}\right)$ and the predictive test quantities $T\left(Y^{\mathrm{rep},l},\omega^{l}\right)$ are computed. The distribution of the predictive test quantities serves as the *reference* distribution, while the distribution of the realized test quantities becomes the *observed* distribution. The posterior predictive p -value (PPP value), a Bayesian counterpart of the classical p -value, is approximated as the proportion of draws where the predictive test quantity is larger than the corresponding realized test quantity. That is,

(7) $$\frac{1}{L}\sum_{l=1}^{L}1\left\{T\left(Y^{\mathrm{rep},l},\omega^{l}\right)>T\left(Y^{\mathrm{obs}},\omega^{l}\right)\right\}.$$

The PPP value indicates the probability that the data-model discrepancy is greater in the replicated data than in the observed data. If the model fits the data well, the PPP value will be near 0.5 (Meng (1994); Sinharay & Stern, 2003). If the PPP value is lower than a nominal significance level α , the hypothesized model is rejected.

### Selection of Optimal Reference Values

In the context of a simulation study, the observed dataset $Y^{\mathrm{obs}}$ described in the previous sections is essentially a replication drawn from a predictive distribution,

(8) $$p\left(Y^{\mathrm{rep}}|H^{\mathrm{true}}\right)=\int_{\omega }p\left(Y^{\mathrm{rep}}|\omega ,H^{\mathrm{true}}\right)p\left(\omega |H^{\mathrm{true}}\right)d\omega$$

where $H^{\mathrm{true}}$ represents the data-generating model. This formulation differs from the posterior predictive distribution (Equation [6]) in that it does not condition on any observed data. The data generation procedure in a simulation study realizes the predictive distribution by sampling true item parameters from a predetermined prior distribution, $p\left(\omega |H^{\mathrm{true}}\right)$ and then sampling data from the data distribution $p\left(Y^{\mathrm{rep}}|\omega ,H^{\mathrm{true}}\right)$ that conditions on the true item parameters and the data-generating model.

Following the data generation, we calibrate the data with a hypothesized model H to obtain a discrepancy measure $T\left(Y^{\mathrm{rep}},\hat{\omega }\right)$ where ω^ is a vector of item parameter estimates under H . If H coincides with $H^{\mathrm{true}}$ , $T\left(Y^{\mathrm{rep}},\hat{\omega }\right)$ is considered drawn from the null distribution of the discrepancy measure. On the contrary, if H does not coincide with $H^{\mathrm{true}}$ , $T\left(Y^{\mathrm{rep}},\hat{\omega }\right)$ is considered drawn from the non-null distribution of the discrepancy measure. Hence, by collecting $T\left(Y^{\mathrm{rep}},\hat{\omega }\right)$ from $H=H^{\mathrm{true}}$ conditions and those from $H\neq H^{\mathrm{true}}$ conditions, we construct an empirical distribution of the discrepancy measures for null and non-null conditions, respectively. Then, we determine reference values (cutoff thresholds) that help infer whether a given discrepancy measure originates from a correctly specified model or a misspecified model.

The diagnostic performance of all possible reference values was evaluated using the ROC curve method. The ROC curve plots the false-positive rate (false alarm rate, or 1 minus specificity) on the x -axis and the true-positive rate (hit rate or sensitivity) on the y -axis across all possible reference values (Pepe, 2003). In the context of item fit diagnostic, sensitivity represents the proportion of misfit items correctly identified as misfit (true-positive rate). Meanwhile, specificity represents the proportion of fit items correctly identified as fit (true-negative rate). An ideal test would have both sensitivity and specificity values of ones, indicating a perfect classification.

The ROC curve provides the basis for determining an optimal reference value, a cutoff threshold that achieves the best balance between sensitivity and specificity. This approach has been proven valuable when gold standards are not available and cutoff values are needed to distinguish between normal and abnormal conditions (e.g., Beck et al., 2019; Nahm, 2022). The appropriate trade-off between sensitivity and specificity depends on their relative importance or benefit. In the context of item fit diagnostic, it is crucial to maximize the true positives by flagging as many misfitting items as possible, while minimizing the false positives to avoid unnecessary reviews or edits of items which can be time-consuming and costly. Therefore, both sensitivity and specificity often play equally important roles in ensuring an efficient and effective diagnostic process, although their relative importance may depend on the specific purpose of item fit evaluation.

To determine the cutoff value that equally weighs sensitivity (Se) and specificity (Sp), we applied the Youden’s J statistic (Youden, 1950) that identifies the cutoff value by maximizing Se+Sp−1 . This criterion reflects the intention of maximizing the correct classification rate, thereby minimizing the misclassification rate. Another widely used approach is minimizing $\sqrt{{\left(1-\mathrm{Se}\right)}^{2}+{\left(1-\mathrm{Sp}\right)}^{2}}$ , the Euclidean distance from the coordinate (0,1) that represents perfect classification. While the Euclidean distance criterion is geometrically appealing, the quadratic terms involved do not directly correspond to the correct classification rate, which may introduce the risk of an increased rate of misclassification (Perkins & Schisterman, 2006). Therefore, the Youden’s J statistic was chosen as the optimization criterion given its clear interpretation in maximizing the correct classification rate.

## Study 1

In Study 1, we implemented the two approaches to interpreting the RMSD values: (a) the PP-PPMC approach to compute their significance values and (b) the cutoff threshold approach to derive reference values for specific data characteristics. Their performances were compared via Type I error rate and power of the PP-PPMC and false-positive and true-positive rates of the reference values.

### Simulation Design

A simulation study was designed to examine the performance of the RMSD under different data conditions. We manipulated three factors: (1) sample size (5,000; 1,000; 500), (2) test length (100; 40; and 20), and (3) the proportion of misfitting items (0.05; 0.1; and 0.2), resulting in a total of 3×3×3=27 conditions. The number of replications varied with sample size to achieve an aggregate sample size of 500,000. That is, the conditions with a sample size of 5,000, 1,000, and 500 were replicated 100, 500, and 1,000 times, respectively. Data generation was conducted using the open-source software R (R Core Team, 2024).

It is worth reiterating that the data generated in this simulation are used in both of the approaches discussed in the previous section, but their role and interpretation differ between the two. In the PP-PPMC approach, each of the simulated datasets was treated as *observed* data that is, $Y^{\mathrm{obs}}$ in Equations (5) to (7). Meanwhile, in the cutoff threshold approach, the simulated data were treated as *replicated* data drawn from the predictive distribution (Equation [8]), where the RMSD values obtained from the simulated data are used to approximate the distribution of the RMSD values under null and non-null conditions.

### Data Generation

The fitting items were generated under the 2PL model. The parameters were randomly sampled from preset distributions, that is, $p\left(\omega |H^{\mathrm{true}}\right)$ in Equation (8). They were selected based on prior literature on item fit evaluation (e.g., Köhler et al., 2020; Sueiro & Abad, 2011) to ensure a realistic spread of parameters. Specifically, the slope parameters were sampled from a lognormal distribution LN(0,0.5) , and the difficulty parameters were sampled from a normal distribution N(0,1) . To avoid extreme item parameters, we excluded outliers that fell outside of the 2.5th and 97.5th percentiles of the distributions from which they were drawn. The lower and upper bounds correspond to 0.25 and 4.00 for the slope parameter and ∓ 1.96 for the difficulty parameter. The population distribution was assumed to be standard normal.

The misfitting items were generated using a logistic function of a monotonic polynomial with a lower asymptote (LMPA; Falk & Cai, 2016a, 2016b) that provides a flexible yet monotonic shape of IRF by introducing lower asymptotes and/or plateaus. The probability of a correct response for item i by person j is

(9) $$P\left(Y_{\mathrm{ij}}=1|\theta_{j},\;\delta_{i},g_{i},\;\omega_{i},\;\alpha_{i},\tau_{i}\right)=g_{i}+\frac{1-g_{i}}{1+\exp\left[-\left\{\delta_{i}+f_{i}\left(\theta_{j}|\omega_{i},\;\alpha_{i},\tau_{i}\right)\right\}\right]}$$

where f is a monotonic polynomial function of order 2k+1 ,

(10) $$f\left(\theta |\omega ,\;\alpha ,\tau \right)=b_{1}\theta +b_{2}\theta^{2}+\ldots +b_{2k+1}\theta^{2k+1},$$

with k being a user-specified nonnegative integer. For k=0 , the model reduces to the 3PL model under which $\exp\left(\omega_{i}\right)$ , $\delta_{i}$ , and $g_{i}$ are interpreted as item slope, intercept, and guessing parameter, respectively. For k≥1 , α and τ are vectors of length k specified to fit the plateau(s) of different length and location. The coefficients $b={\left(b_{1},b_{2},\ldots ,b_{2k+1}\right)}^{\prime }$ are complicated functions of ω,α, and τ as detailed by Falk and Cai (2016a).

**Figure 1.**
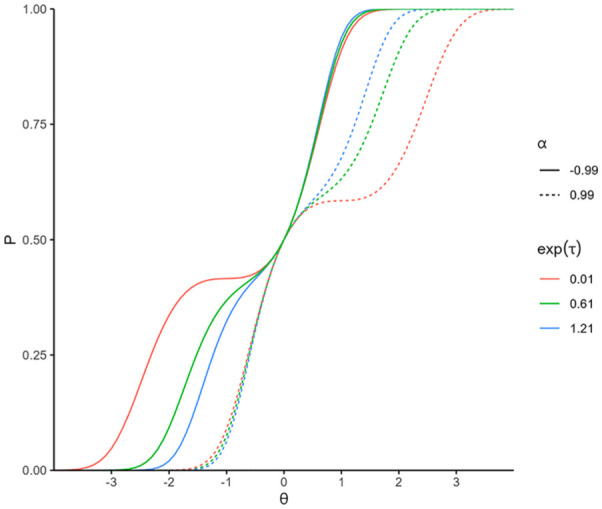
Example of item response functions for misfitting items. *Note*. The item response functions are generated using the LMPA with parameter values of k=1 , g=0 , ω=0 , and δ=0 , while varying in their α and τ values.

To simulate the IRFs of misfitting items, we conducted a preliminary analysis to identify the combinations of parameters in the LMPA that yield non-negligible degrees of item misfit. The order of f (i.e., 2k+1 ) was set to 0 or 3, as achieved by k=0 or 1 . For k=0 , $g_{i}$ varied from 0.1 to 0.3 in increments of 0.1, and $\omega_{i}$ was taken as the 10th to 90th percentiles in increments of 10% of a standard normal distribution. The resulting $\exp\left(\omega_{i}\right)$ ranged from 0.27 to 3.67, which covers a plausible range of item slope. Assuming that item difficulty parameters are typically drawn from a standard normal distribution (e.g., Köhler et al., 2020; Sueiro & Abad, 2011), the intercept—as a product of item slope and difficulty—approximately ranges from −1.6 to 1.6. Hence, $\delta_{i}$ was set to vary from −1.6 to 1.6 in increments of 0.4. These resulted in a total of 3×9×9=243 combinations of parameters. For k=1 , in addition to $g_{i},\;\omega_{i}$ , and $\delta_{i}$ parameter values specified for k=0 , the guessing parameter $g_{i}$ was allowed to take a value of zero, resulting in an IRF with plateaus but without lower asymptote. As parameters generating the plateaus, $\alpha_{i}$ varied from −0.99 to 0.99 in increments of 0.22, and $\exp\left(\tau_{i}\right)$ varied from 0.01 to 1.21 in increments of 0.2. These values were chosen to encompass the simulated conditions of Falk and Cai (2016a). The resulting number of combinations was 4×9×9×10×7=22,680 . Examples of IRFs generated by the LMPA are presented in Figure 1. It is illustrated that $\alpha_{i}$ determines the location of the plateau, while $\tau_{i}$ governs its length.

For each of the possible combinations, we compared the true IRF generated by the LMPA with the IRF of the 2PL model that best approximates the true IRF. The approximating 2PL model was obtained by fitting a 2PL model to the true IRF with population density (that is assumed to be a standard normal in this study) as weights. Then, we excluded the cases in which one or more of the parameters of the fitted 2PL model are extreme, that is, beyond the plausible range of item parameters. The fitted parameter was considered extreme if it fell outside two standard deviations from the mean of the distribution (i.e., LN(0,0.5) for the slope parameters and N(0,1) for the difficulty parameters, consistent with the generation of fitting items). We then calculated the RMSD to quantify the difference between the true IRF and its approximation by 2PL model. The resulting RMSD values ranged from 0.000 to 0.129, with a mean of 0.045 and standard deviation of 0.022. Since these RMSD values were not affected by sampling or estimation errors—hence the term *population* RMSD values (e.g., Köhler et al., 2020; Robitzsch, 2022)—they were interpretable as effect sizes. Following Köhler et al. (2020), the combinations with the RMSD values above 0.05 were deemed to be generating medium to large misfit. A total of 7,612 combinations satisfied the condition. To generate misfitting items, parameters of the LMPA were randomly drawn from the pool of combinations that produced medium to large misfit.

### Parameter Estimation

The calibration of 2PL models was conducted using flexMIRT^®^ (Cai, 2024). Item parameters were estimated using the Bock–Aitkin EM algorithm (Bock & Aitkin, 1981), and the covariances of the item parameters were computed by the supplemented EM algorithm (Cai, 2008). Quadrature points used for calibration and item fit analysis were 81 equally spaced points ranging from −4 to 4.

### PP-PPMC Implementation

Following the parameter estimation, L=100 sets of item parameters were drawn from their posterior distribution, approximated by a multivariate normal distribution. The sampled item parameters were used to generate replicated data using flexMIRT^®^ (Cai, 2024). The realized and predictive test quantities were computed and compared in R (R Core Team, 2024).

### Selection of Optimal Reference Values

The magnitude of the RMSD is known to depend heavily on data characteristics such as sample size and test length, while remaining robust to variations in the proportion of misfitting items (Köhler et al., 2020, 2021; Robitzsch, 2022; Sueiro & Abad, 2011). Hence, the ROC curve was generated separately for different combinations of sample size and test length. That is, the distribution of discrepancy measures was conditioned on some fixed features of data (Rubin, 1984). For each combination of sample size and test length (e.g., sample size of 5,000 and test length of 100), the ROC curve was constructed as follows: First, the RMSD values were computed for all of the simulated datasets; second, equally spaced values with a minimal grid size (e.g., 0.001) within the range of observed RMSD values were considered as candidate reference values; third, each of the candidate reference values was applied to the observed RMSD values, and the resulting false-positive and true-positive rates were plotted as points; finally, the points were connected to form a line, that is, the ROC curve.

**Figure 2.**
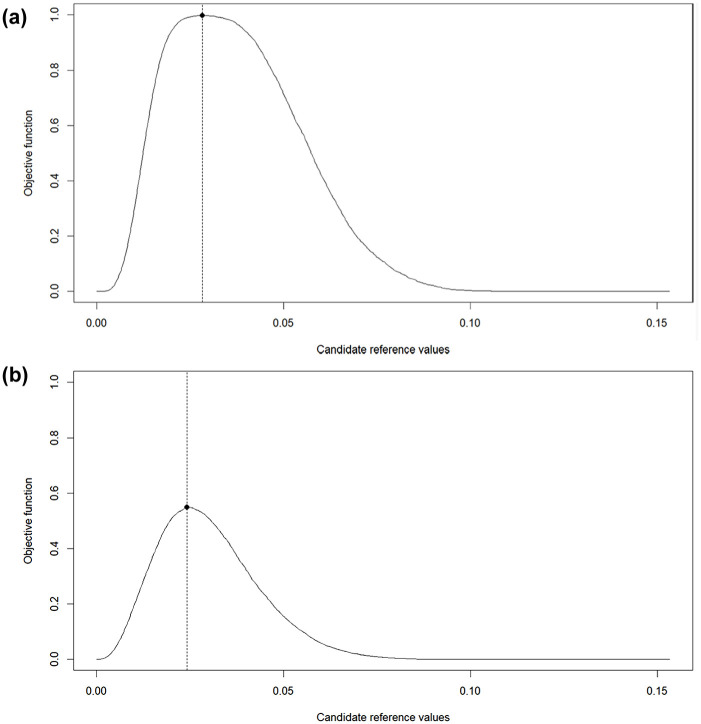
Example of reference value optimization. (a) Sample size = 5,000; test length = 100. (b) Sample size = 500; test length = 20. *Note*. The points represent local maxima of the objective function.

Based on the ROC curves, the Youden’s J statistic criterion was applied to identify —for each combination of sample size and test length—the reference Value that achieves an optimal balance between sensitivity and specificity. Examples of the optimization procedure are presented in Figure 2. The reference value that maximizes the objective function, indicated by a dot, is selected as the optimal value. Figure 2(a) illustrates the condition with the largest sample size and the longest test length, and Figure 2(b) depicts the condition with the smallest sample size and the shortest test length. The optimized value in Figure 2(a) achieves a near-perfect classification with sensitivity and specificity of ones, while the optimized value in Figure 2(b) achieves less accurate classification. These examples suggest that the diagnostic performance of an optimal reference value is constrained by the amount of information available in the data.

### Results

The results of implementing the PP-PPMC for statistical testing and the ROC method for establishing reference values are presented in the following.

#### Type I Error Rate and Power

**Figure 3.**
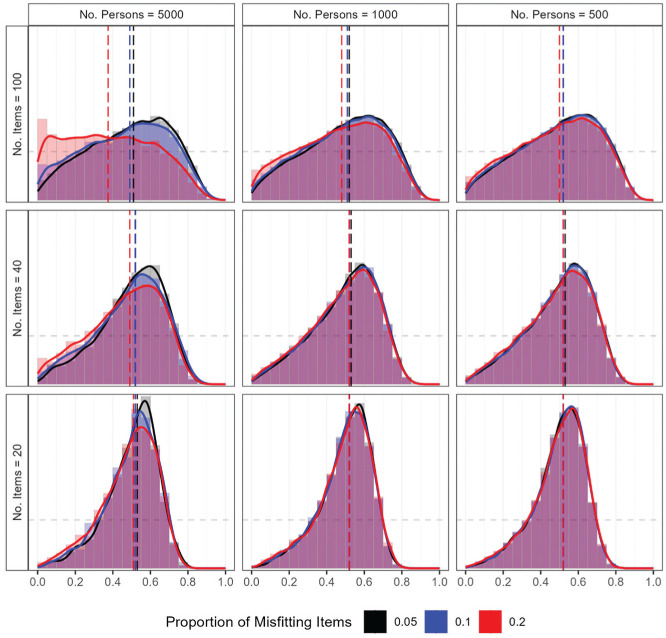
Study 1: Distribution of PPP values under null conditions. *Note*. The gray horizontal dashed line represents a uniform distribution. The vertical lines indicate the median of the PPP values, which the color differs by the proportion of misfitting items. PPP = posterior predictive p -value.

The performance of the RMSD under null conditions was first examined via the distribution of PPP values for fitting items. Figure 3 presents the distribution of PPP values, with each panel corresponding to a specific combination of sample size and test length. In each panel, separate distributions are shown for different proportions of misfitting items. The median PPP values were consistently around 0.5, except when the proportion of misfitting items was the highest (i.e., 0.2) under the largest sample size (i.e., 5,000) and the longest test length (i.e., 100). The distributions were closest to uniform when the number of items was largest (i.e., 100) and became increasingly concentrated around 0.5 as the number of items decreased. The pattern reflects the tendency of PPP values to have a tighter concentration around 0.5 when less information is available in the data (e.g., Gilbride & Lenk, 2010). Furthermore, when the sample size was largest (i.e., 5,000), as the proportion of misfitting items increased, the PPP values were less concentrated around the center, with more PPP values near zero. Especially when the largest sample size was combined with the longest test length, the highest proportion of misfitting items (i.e., 0.2) resulted in a noticeable peak of PPP values near zero.

The resulting Type I error rate and power are summarized in Table 1. The Type I error rates were mostly below the nominal level of 0.05, except when the proportion of misfitting items was 0.2 with 5,000 persons and 100 items, where the false alarm rate was mildly inflated to 0.08. The power was nearly one (i.e., all misfitting items were detected) when the sample size was 5,000. For smaller sample sizes, the power decreased as the number of items decreased. Specifically, the power ranged from 0.54 to 0.91 with the sample size of 1,000, and from 0.23 to 0.65 with the sample size of 500. Notably, no systematic differences in power were observed across varying proportions of misfitting items.

**Table 1.** Study 1: Results of Statistical Significance Testing and Cutoff Threshold Application.

Proportion of misfitting items	Samplesize	Test length	PP-PPMC approach		Cutoff threshold approach	
			Type Ierror rate	Power	False-positiverate	True-positiverate
0.05	5,000	100	0.02	1.00	0.00	1.00
		40	0.01	1.00	0.00	0.99
		20	0.00	1.00	0.01	1.00
	1,000	100	0.02	0.91	0.05	0.93
		40	0.01	0.82	0.07	0.90
		20	0.00	0.54	0.11	0.86
	500	100	0.02	0.65	0.15	0.83
		40	0.01	0.48	0.16	0.80
		20	0.00	0.24	0.18	0.74
0.10	5,000	100	0.04	1.00	0.00	1.00
		40	0.01	1.00	0.00	1.00
		20	0.00	0.97	0.01	0.97
	1,000	100	0.02	0.90	0.06	0.92
		40	0.01	0.82	0.07	0.92
		20	0.00	0.54	0.11	0.83
	500	100	0.02	0.64	0.16	0.82
		40	0.01	0.50	0.16	0.82
		20	0.00	0.23	0.18	0.73
0.20	5,000	100	0.08	1.00	0.00	1.00
		40	0.03	1.00	0.00	1.00
		20	0.01	0.99	0.02	0.99
	1,000	100	0.03	0.89	0.08	0.90
		40	0.01	0.80	0.08	0.90
		20	0.00	0.54	0.12	0.85
	500	100	0.02	0.61	0.18	0.81
		40	0.01	0.47	0.17	0.79
		20	0.00	0.24	0.18	0.73

#### Optimized Reference Values

**Figure 4.**
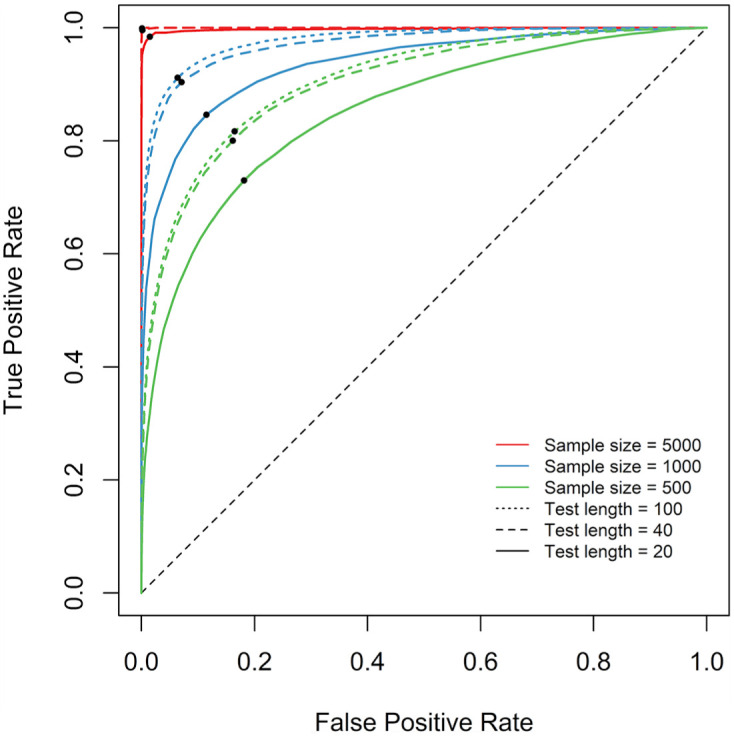
Study 1: ROC curves and optimized reference values. *Note*. The black dots indicate the false-positive and true-positive rates achieved by optimal reference values. ROC = receiver operating characteristic.

The ROC curves that represent the diagnostic performances of the RMSD under different data characteristics are presented in Figure 4. Each point marks the coordinate of the false-positive rate and true-positive rate under the optimal reference value. The condition with the sample size of 5,000 and the test length of 100, illustrated in Figure 2(a), is represented by the red dotted line that lies furthest away from the diagonal and reaches the point of perfect classification (i.e., [0,1]). This indicates that, when sufficient information is available in the data, the distribution of the RMSD values for fitting items show minimal overlap with that for misfitting items, thereby allowing the optimized cutoff value to achieve near-perfect classification. By contrast, the with the sample size of 500 and the test length of 20, illustrated in Figure 2(b), is represented by the green solid line that lies closest to the diagonal. This suggests that, when there is limited amount of information in the data, the distributions of the RMSD values for fitting and misfitting items overlap considerably, thereby limiting the classification performance of the optimized cutoff value.

The optimized reference values are presented in the first three columns of Table 2. They tend to decrease as the test length decreases and as the sample size increases, while this effect of test length decreases as the sample size increases. Such a pattern is in alignment with previous findings on the dependence of the RMSD on test length and sample size (e.g., Köhler et al., 2020, 2021; Sueiro & Abad, 2011). An analytical discussion of the sources of bias in the RMSD attributable to estimation error can be found in Robitzsch (2022).

**Table 2.** Optimized Reference Values by Sample Size and Test Length.

	Sample size					
Test length	500	1,000	5,000	10,000	20,000	50,000
20	0.025	0.020	0.013	-	-	-
40	0.036	0.030	0.021	-	-	-
100	0.051	0.042	0.028	0.024	[0.022, 0.031]	[0.018, 0.028]
200	-	-	-	0.030	[0.027, 0.035]	[0.020, 0.038]
400	-	-	-	0.036	0.036	[0.027, 0.037]

*Note*. The lower bound values within the interval are recommended for practical use to maximize true-positive rates in the presence of missing data.

The false-positive and true-positive rates of the suggested reference values are summarized in Table 1. Compared to the statistical testing via the PP-PPMC, applying the cutoff thresholds generally resulted in higher false-positive rates but also higher true-positive rates. For instance, under the sample size of 5,000 and test length of 100 or 40, the cutoff threshold approach yielded lower false-positive rates while also maintaining near-perfect true-positive rates. That is, while the PP-PPMC approach resulted in an inflated Type I error rate under a higher proportion of misfitting items, the cutoff thresholds approach consistently yielded a near-zero Type I error rate, regardless of the proportion of misfitting items. In conditions with smaller sample sizes, as the proportion of misfitting items increased, the false-positive rate increased and true-positive rate decreased, but only to a marginal degree. This result is consistent with the previous findings that the magnitude of the RMSD is largely unaffected by the proportion of misfitting items (e.g., Köhler et al., 2020; Sueiro & Abad, 2011). Overall, the cutoff threshold approach outperformed the PP-PPMC approach in that the improvement in the true-positive rates outweighed the increase in the false-positive rates, while exhibiting robustness to varying proportions of misfitting items.

## Study 2

We conducted an additional simulation to further generalize the reference values to larger numbers of persons and items. We also examined the performance of the reference values under data missingness which is prevalent in large-scale assessments with a relatively large number of items and persons. The parameter estimation and the selection of optimal reference values followed the same procedure as in Study 1.

### Simulation Design

We manipulated four factors: (1) sample size (50,000; 20,000; and 10,000), (2) test length (400; 200; and 100), (3) the proportion of misfitting items (0.05; 0.1; and 0.2), and (4) the proportion of missing responses per person (0; 0.4; and 0.8), resulting in a total of 3×3×3×3=81 conditions. The number of replications varied with sample size to achieve an aggregate sample size of 2,000,000. That is, the conditions with the sample size of 50,000, 20,000, and 10,000 were replicated 40, 100, and 200 times, respectively. The data generation was conducted using the open-source software R (R Core Team, 2024). It is important to note that only the conditions with no data missingness (i.e., proportion of missing responses set to zero) were used to derive optimized reference values, and those with missingness were used to examine the performance of the optimized reference values when moderate to high degree of data missingness is introduced.

### Data Generation

The fitting and misfitting items were generated using the same procedure as in Study 1. They were generated under 2PL and the LMPA (Falk & Cai, 2016a, 2016b) models, respectively. For conditions with non-zero proportions of data missingness, the assigned proportion of missingness was imposed on each person’s responses to generate random missingness.

### Results

The results are summarized in three parts. First, we discuss the impact of the manipulated factors on the magnitude and dispersion of the RMSD values, with special emphasis on the impact of missing data, along with its interaction with other manipulated factors that was not addressed in Study 1. Then, we present the reference values that were optimized for data without missing responses, where we also discuss the impact of missing responses on the performance of the optimized reference values. Finally, we utilize the optimized reference values from both studies (Study 1 and Study 2) to fit a model that can predict optimal reference values for conditions that were not included in the studies.

#### Descriptive Statistics of RMSD Values

**Figure 5.**
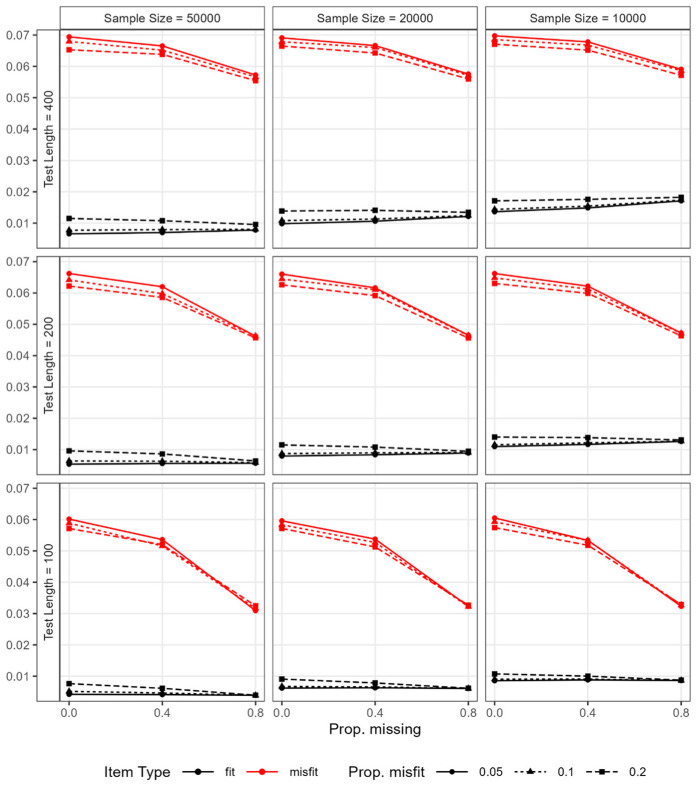
Study 2: Average RMSD values for fitting and misfitting items by condition. *Note*. RMSD = root mean squared deviation.

The descriptive statistics of the RMSD values for fitting and misfitting items are presented in Tables 3 and 4, respectively. Figure 5 illustrates the average RMSD values for fitting and misfitting items by condition. The mean and variance of the RMSD values for fitting items were only minimally impacted by the missingness. The nearly flat black lines in Figure 5 highlight that the mean RMSD values remained fairly stable across varying proportions of missing data. The most impacted condition occurred under the combination of the largest sample size, the shortest test length, and the highest proportion of misfitting items (i.e., sample size = 50,000; test length = 100; proportion of misfitting items = 0.2). In this case, the average RMSD value for fitting items was 0.008, 0.006, and 0.004 when the proportion of missing responses was 0, 0.4, and 0.8, respectively. Hence, we concluded that the magnitude for RMSD values for fitting items is negatively affected by data missingness, though by a marginal degree, when the ratio of sample size to test length is large (e.g., 500 or higher) and the proportion of misfitting items is high (e.g., 0.2 or higher). By contrast, the mean and variance of the RMSD values for misfitting items were both negatively impacted by data missingness. That is, as a larger proportion of data was missing, the mean RMSD values decreased—as signified by the declining red lines in Figure 5—and the variance of RMSD values also decreased.

**Table 3.** Study 2: Descriptive Statistics of RMSD values for Fitting Items.

Proportion ofmisfitting items	Samplesize	Testlength	Proportion of missing responses					
			0.0		0.4		0.8	
			*M*	*SD*	*M*	*SD*	*M*	*SD*
0.05	50,000	400	0.007	0.002	0.007	0.002	0.008	0.003
		200	0.005	0.002	0.006	0.002	0.006	0.002
		100	0.004	0.001	0.004	0.002	0.004	0.002
	20,000	400	0.010	0.003	0.011	0.003	0.012	0.004
		200	0.008	0.002	0.008	0.003	0.009	0.004
		100	0.006	0.002	0.006	0.002	0.006	0.003
	10,000	400	0.014	0.004	0.015	0.004	0.017	0.006
		200	0.011	0.003	0.012	0.004	0.013	0.005
		100	0.009	0.003	0.009	0.003	0.009	0.004
0.10	50,000	400	0.008	0.002	0.008	0.002	0.008	0.003
		200	0.006	0.002	0.006	0.002	0.006	0.002
		100	0.005	0.002	0.005	0.002	0.004	0.002
	20,000	400	0.011	0.003	0.011	0.003	0.012	0.005
		200	0.009	0.003	0.009	0.003	0.009	0.004
		100	0.007	0.002	0.007	0.003	0.006	0.003
	10,000	400	0.014	0.004	0.015	0.005	0.017	0.006
		200	0.012	0.004	0.012	0.004	0.013	0.005
		100	0.009	0.003	0.009	0.004	0.009	0.004
0.20	50,000	400	0.011	0.003	0.011	0.003	0.010	0.003
		200	0.010	0.003	0.009	0.003	0.006	0.003
		100	0.008	0.003	0.006	0.002	0.004	0.002
	20,000	400	0.014	0.004	0.014	0.004	0.013	0.005
		200	0.011	0.003	0.011	0.004	0.009	0.004
		100	0.009	0.003	0.008	0.003	0.006	0.003
	10,000	400	0.017	0.005	0.018	0.005	0.018	0.007
		200	0.014	0.004	0.014	0.005	0.013	0.006
		100	0.011	0.004	0.010	0.004	0.009	0.004

**Table 4.** Study 2: Descriptive Statistics of RMSD Values for Misfitting Items.

Proportion ofmisfitting items	Samplesize	Testlength	Proportion of missing responses					
			0.0		0.4		0.8	
			*M*	*SD*	*M*	*SD*	*M*	*SD*
0.05	50,000	400	0.069	0.015	0.067	0.015	0.057	0.013
		200	0.066	0.014	0.062	0.013	0.046	0.012
		100	0.060	0.013	0.054	0.013	0.031	0.009
	20,000	400	0.069	0.014	0.067	0.014	0.058	0.014
		200	0.066	0.015	0.062	0.014	0.047	0.012
		100	0.060	0.013	0.054	0.013	0.032	0.009
	10,000	400	0.070	0.015	0.068	0.015	0.059	0.014
		200	0.066	0.014	0.062	0.013	0.047	0.013
		100	0.061	0.014	0.053	0.013	0.032	0.010
0.10	50,000	400	0.068	0.015	0.065	0.014	0.057	0.012
		200	0.064	0.014	0.060	0.013	0.046	0.011
		100	0.059	0.014	0.052	0.012	0.032	0.009
	20,000	400	0.068	0.015	0.066	0.014	0.057	0.013
		200	0.064	0.014	0.061	0.014	0.046	0.012
		100	0.058	0.013	0.053	0.013	0.032	0.010
	10,000	400	0.069	0.015	0.067	0.015	0.059	0.014
		200	0.065	0.014	0.061	0.014	0.047	0.013
		100	0.059	0.014	0.053	0.013	0.033	0.010
0.20	50,000	400	0.065	0.014	0.064	0.014	0.055	0.013
		200	0.062	0.014	0.059	0.013	0.046	0.012
		100	0.057	0.013	0.052	0.012	0.032	0.009
	20,000	400	0.066	0.014	0.064	0.014	0.056	0.013
		200	0.063	0.014	0.059	0.013	0.046	0.012
		100	0.057	0.013	0.051	0.012	0.033	0.010
	10,000	400	0.067	0.015	0.065	0.015	0.057	0.014
		200	0.063	0.014	0.060	0.014	0.046	0.013
		100	0.057	0.013	0.052	0.012	0.033	0.011

As a result of these changes in the RMSD values, the distance between the average RMSD values for fitting and misfitting items decreased as the level of missingness increased, and this impact of missingness was larger under shorter test lengths (e.g., 100). Moreover, as the proportion of misfitting items increased, the distance between the average RMSD values slightly decreased, although the magnitude of this influence was small and further diminished to near zero as the proportion of missing responses increased. In other words, the impact of the proportion of misfitting items became even more negligible as the proportion of missing responses was larger.

#### Optimized Reference Values

Among the simulated conditions, only those with no missing responses were used to optimize the reference values. This choice was made primarily to maintain consistency with Study 1, where no missing responses were presumed. The same ROC curve analysis as in Study 1 was applied to identify reference values that maximize the sum of specificity and sensitivity, following the Youden’s J criterion. The optimized values are presented in the last three columns of Table 2. They achieved the false-positive and true-positive rates of 0.00 and 1.00, that is, perfect classification, within each combination of sample size and test lengths. It is notable that, when the sample size is large (e.g., 20,000 or larger) and the ratio of sample size to test length is high (e.g., 100 or higher), there were more than one candidate cutoff value that achieved the optimal classification performance. For instance, with a sample size of 50,000 and a test length of 400, the candidate cutoff values ranging from 0.027 to 0.037 all achieved the same classification performance of 0.00 false-positive rate and 1.00 true-positive rate. This result is due to the distributions of the RMSD values for fitting and misfitting items exhibiting minimal overlap when abundant information is available in a dataset (see Figure 5).

When the optimized reference values form an interval rather than a single point estimate, we suggest using the lower bound of the interval. This recommendation accounts for potential presence of data missingness, which is a common scenario in large-scale assessments. As discussed in the previous subsection, the average RMSD values for misfitting items tend to decrease as the proportion of missing responses increases, whereas the average RMSD values for fitting items remain stable. Therefore, adopting the lower bound of the optimal interval helps guard against the loss of power in detecting misfitting items in the presence of missing responses. The result of applying the lower bound of the interval as a cutoff is summarized in Table 5. As noted earlier, the conditions with no missing data were used to optimize the cutoff thresholds, so their false-positive and true-positive rates are zero and one, respectively. When 40% of item responses were missing, the performance of the cutoff thresholds was unaffected in terms of the false-positive and true-positive rates remaining zero and one, respectively. When 80% of item responses were missing, however, the true-positive rates decreased for smaller sample sizes (e.g., 10,000 or 20,000) and shorter test length (e.g., 100), while the false-positive rates remained zero. This result is as expected, as the average RMSD values for fitting items remained stable, while the average RMSD values for misfitting items decreased with data missingness. The most impacted condition occurred under the smallest sample size (i.e., 10,000) and the shortest test length (i.e., 100), where the true-positive rate ranged from 0.75 to 0.78, depending on the proportion of misfitting items. Except for this condition, the true-positive rate was 0.83 or higher, showing that the suggested reference values performed sufficiently well even under severe levels of data missingness.

**Table 5.** Study 2: Result of Cutoff Threshold Application.

Proportion of misfitting items	Samplesize	Testlength	Proportion of missing responses					
			0.0		0.4		0.8	
			False-positiverate	True-positiverate	False-positiverate	True-positiverate	False-positiverate	True-positiverate
0.05	50,000	400	0.00	1.00	0.00	1.00	0.00	1.00
		200	0.00	1.00	0.00	1.00	0.00	1.00
		100	0.00	1.00	0.00	1.00	0.00	0.91
	20,000	400	0.00	1.00	0.00	1.00	0.00	0.97
		200	0.00	1.00	0.00	1.00	0.00	0.96
		100	0.00	1.00	0.00	1.00	0.00	0.86
	10,000	400	0.00	1.00	0.00	1.00	0.00	0.97
		200	0.00	1.00	0.00	1.00	0.00	0.91
		100	0.00	1.00	0.00	1.00	0.00	0.75
0.10	50,000	400	0.00	1.00	0.00	1.00	0.00	1.00
		200	0.00	1.00	0.00	1.00	0.00	1.00
		100	0.00	1.00	0.00	1.00	0.00	0.93
	20,000	400	0.00	1.00	0.00	1.00	0.00	0.97
		200	0.00	1.00	0.00	1.00	0.00	0.96
		100	0.00	1.00	0.00	1.00	0.00	0.83
	10,000	400	0.00	1.00	0.00	1.00	0.00	0.96
		200	0.00	1.00	0.00	1.00	0.00	0.92
		100	0.00	1.00	0.00	1.00	0.00	0.78
0.20	50,000	400	0.00	1.00	0.00	1.00	0.00	1.00
		200	0.00	1.00	0.00	1.00	0.00	1.00
		100	0.00	1.00	0.00	1.00	0.00	0.95
	20,000	400	0.00	1.00	0.00	1.00	0.00	0.95
		200	0.00	1.00	0.00	1.00	0.00	0.95
		100	0.00	1.00	0.00	1.00	0.00	0.85
	10,000	400	0.00	1.00	0.00	1.00	0.01	0.94
		200	0.00	1.00	0.00	1.00	0.00	0.90
		100	0.00	1.00	0.00	1.00	0.00	0.77

#### Generalization of Recommended Reference Values

To further generalize the recommended cutoff values from Study 1 and Study 2 to conditions that were not included in the simulation conditions, we fitted a model to predict them based on sample size and test length. This approach, known as response surface analysis (Box, 1954), involves fitting a polynomial equation to experimental data. Its application can be found in analytical chemistry (e.g., Bezerra et al., 2008) as well as educational psychology (e.g., Zhao et al., 2024). The analysis was performed by regressing the optimal cutoff values on sample size and test length. Let Z denote the outcome variable (i.e., the suggested cutoff values), and N and n represent sample size and test length, respectively. Note that $\sqrt{N}$ and $\sqrt{n}$ were used as predictors instead of N and n , respectively, to regulate the size of the coefficients. We evaluated a series of regression models using the first-, second-, and third-order combinations of the predictors.

Table 6 summarizes the predictors included in each model, along with the corresponding variance explained (i.e., $R^{2}$ and adjusted $R^{2}$ ). We started with the main effects of $\sqrt{N}$ and $\sqrt{n}$ as predictors (Model 1), which resulted in an insufficient amount of variance explained, especially after accounting for the number of predictors (i.e., adjusted $R^{2}$ = .586). As we added the main effects of N and n (Model 2), the amount of variance explained considerably increased (adjusted $R^{2}$ = .879). Including the main effects of $N^{3/2}$ and $n^{3/2}$ (Model 4) further improved the variance explained (adjusted $R^{2}$ = .942). We additionally examined the explanatory power of interaction terms such as $n^{1/2}N^{1/2}$ in Model 3, $n^{1/2}N$ in Model 5, $nN^{1/2}$ in Model 6, and nN in Model 7. However, these interaction terms resulted in a marginal increase in $R^{2}$ and a decrease in adjusted $R^{2}$ , indicating potential overfitting. Based on these results, Model 4 was selected as a final model that strikes a balance between goodness-of-fit and parsimony as indicated by the highest adjusted $R^{2}$ .

**Table 6.** Explained Variance of Polynomial Regression Models.

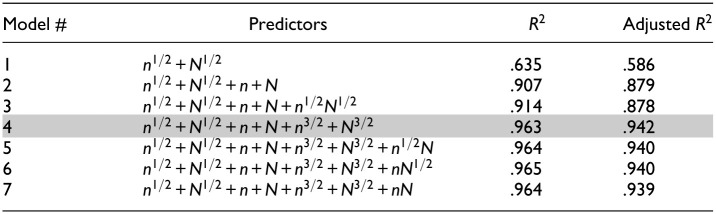

*Note*. *n* and *N* denote the number of items and the sample size, respectively. The shaded cells indicate the polynomial regression model selected as the final model.

**Figure 6.**
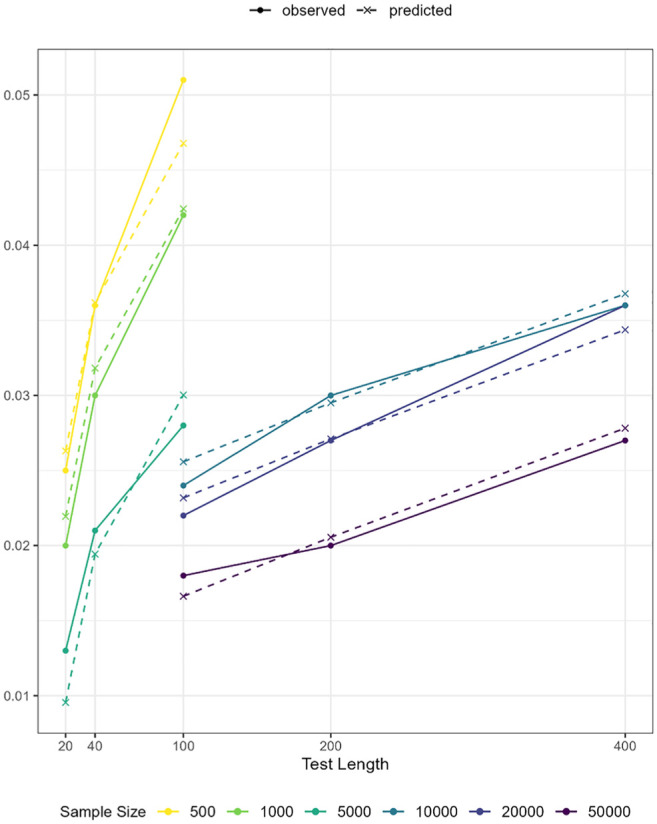
Observed and predicted reference values by sample size and test length.

In the final model (Model 4), all coefficients for the predictors were statistically significant, as shown in Table 7. In Figure 6, the model-predicted reference values (dashed lines) are overlaid with the observed reference values (solid lines), showing close overlap. Notably, the residuals (observed minus fitted values) are relatively large at the lowest and the highest ends of the observed or predicted values. Specifically, the residual was 0.013 − 0.010 = 0.003 under the condition of 5,000 persons and 20 items, and 0.051 − 0.047 = 0.004 under the condition of 500 persons and 100 items. The two cases, respectively, correspond to the conditions with the highest and lowest ratio of sample size to test length in Study 1. The diagnostic plots in Supplemental Materials also highlight these two cases as influential points. Such patterns were expected as residuals tend to be larger at the extremes of the outcome range, and the absolute magnitude of these residuals remained small. Therefore, we conclud that this model can be used to compute reference values tailored to the specific sample size and test length of the dataset at hand.

**Table 7.** Coefficients of the Selected Polynomial Regression Model (Model 4).

Predictor	Estimate	*SE*	p
Intercept	−0.0025542254	0.0085534610	0.771
$n^{1/2}$	0.0128772104	0.0025741814	0.000
$N^{1/2}$	−0.0006866889	0.0001152466	0.000
n	−0.0008641079	0.0002418713	0.004
N	0.0000044058	0.0000011980	0.004
$n^{3/2}$	0.0000202367	0.0000067004	0.012
$N^{3/2}$	−0.0000000099	0.0000000033	0.013

*Note*. The estimates and corresponding standard errors were rounded to 10 decimal places.

## Empirical Example

In this section, we demonstrate an application of the RMSD using an empirical example collected from a large-scale computer-based assessment on Calculus. The dataset consists of item responses of 13,217 examinees to 132 dichotomously scored items. A 2PL model was hypothesized for all items. The average proportion of missing responses per person was 69%. Based on the model selected in the Generalization of Recommended Reference Values section (Model 4 of which coefficients are presented in Table 7), the suggested reference value tailored for this dataset was 0.026. As a result of applying the cutoff, a total of 41 items were flagged for meaningfully deviating from the hypothesized model.

**Figure 7.**
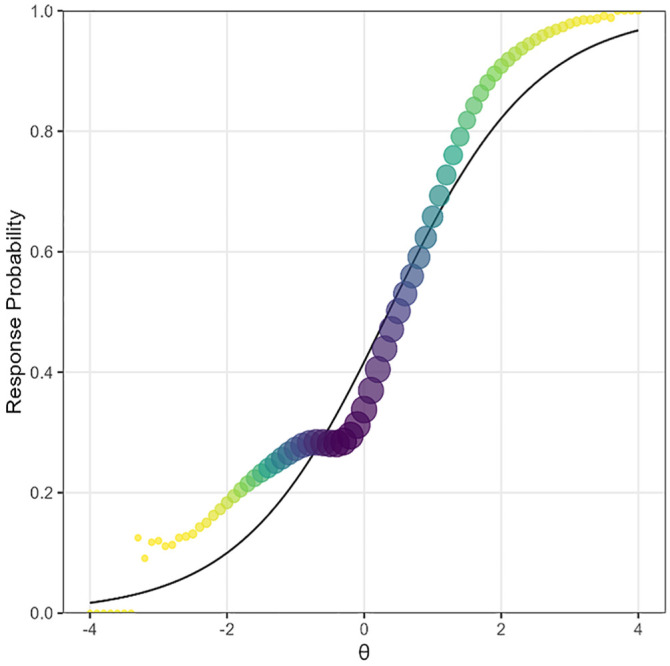
Empirical example of observed and expected item response functions.

All misfitting items exhibited patterns that warrant further scrutiny, and their IRFs often exhibited one or more plateaus. As an example, Figure 7 visualizes the discrepancies between observed and expected IRFs found in one of the flagged items. The solid line represents the expected or model-implied response probabilities of correct response, and the points indicate observed probabilities of correct response. The size of each point corresponds to the weight assigned by the standard normal density. This item with the RMSD value of 0.057 exhibited a plateau around θ value of −1, which is accompanied by a steeper slope at higher (\theta) values.

## Summary and Discussions

### Summary

Posterior expectations, otherwise known as pseudocounts, provide a powerful and accessible foundation for item fit analysis. They eliminate the need to categorize examinees into potentially imprecise subgroups, while fully incorporating the uncertainties of the latent variable estimates. Even in small sample sizes or under substantial data missingness, they ensure a smooth distribution of examinees across the continuum. Moreover, they are natural by-products of the Bock–Aitkin EM algorithm, a standard estimation method for IRT models, which makes pseudocount-based item fit indices highly accessible to users of IRT models. Given these advantages, the RMSD based on posterior expectations, along with corresponding visualizations, has been widely adopted. This study enhanced the interpretability of the RMSD by implementing two approaches the statistical significance testing via the PP-PPMC and the optimization of reference values (cutoff thresholds) using the ROC curve analysis.

In Study 1, we showcased that the PP-PPMC (Lee et al., 2016) can be used to compute statistical significance levels for the RMSD. The PP-PPMC inherently accounted for the estimation and sampling errors, which resolved the key obstacles in identifying the null distributions of the RMSD. As a result, the Type I error rates were well-controlled, and the power was moderate to high in most cases. However, in conditions with a small sample size (e.g., 500) and a short test length (e.g., 20), the power considerably declined while the Type I error rate remained below the nominal level. As an alternative approach, we implemented the ROC curve analysis (Metz, 1978) to empirically derive reference values that achieve the best balance of the false-positive and true-positive rates. Applying the optimized reference values resulted in both higher false-positive rates and higher true-positive rates, compared to the PP-PPMC approach, especially under data-limited scenarios with smaller sample sizes and/or shorter test lengths. Overall, the cutoff threshold approach offered significant improvement in the true-positive rates, albeit with a slight increase in the false-positive rates.

In Study 2, we extended the ROC curve analysis to conditions with larger sample sizes and longer test lengths, reflecting scenarios typical of large-scale assessments. The optimized reference values achieved a near-perfect classification under complete datasets. In the presence of missing responses, the true-positive rates moderately declined, while the false-positive rates remained close to zero. To further generalize the suggested reference values beyond the simulated conditions, we employed the response surface analysis (Box, 1954). Specifically, a polynomial regression model was fitted by regressing the suggested reference values from Study 1 and Study 2 on the linear, quadratic, and cubic effects of test length and sample size. The model allows practitioners to compute benchmark values tailored to the characteristics of their datasets.

### Key Contributions

This study makes several key contributions. First, we employed the PP-PPMC (Lee et al., 2016) to implement a pseudo-Bayesian model checking approach for item fit diagnostics in a frequentist framework. Second, we used the ROC analysis (Metz, 1978) to empirically derive reference values optimized for specific combinations of test length and sample size—two factors well known for their influence on the magnitude of RMSD. This highlights the necessity of selecting reference values based on data-specific conditions. Recent work by von Davier and Bezirhan (2023) similarly suggested identifying misfitting items by selecting outliers in a dataset-specific distribution of the RMSD values. Condition-specific reference values have been highlighted in other contexts as well (e.g., McNeish & Wolf, 2021). Third, we evaluated the performance of the optimized cutoff thresholds under varying degrees of data missingness. As missing responses are introduced into a dataset, the RMSD values of fitting items largely remained stable, while the RMSD values of misfitting items noticeably decreased, particularly when the test length was shorter. Consequently, applying the optimized cutoffs resulted in moderately reduced true-positive rates while maintaining stable, near-zero false-positive rates. Fourth, we presented a prediction model that predicts the suggested reference values based on test length and sample size, enabling practitioners to compute tailored thresholds for the RMSD with ease. This approach can be extended seamlessly to other item fit measures, such as RMSDs with alternative weights (e.g., Joo et al., 2024) or those incorporating nonparametric or semiparametric methods for computing observed probabilities (e.g., Douglas & Cohen, 2001; Köhler et al., 2021; Lee et al., 2009).

### Limitations

This study has limitations that constrain the interpretation of its results and warrant further research. First, it is important to examine whether the RMSD performs similarly under conditions beyond those simulated in this study. Potential factors that may influence the performance of the RMSD include calibration model (e.g., 3PL model), type of items (e.g., items with more than two score categories), type of item misfit (e.g., nonmonotone IRF), systematic missing pattern (i.e., missing not at random), or violation of standard IRT model assumptions (e.g., unidimensionality, local independence, and normal population density). Since the optimality of the recommended reference values is contingent upon the simulated conditions, these reference values may need to be re-evaluated in other contexts.

Second, this study evaluated the performance of the RMSD in the context of testing whether a hypothesized model (e.g., 2PL model) adequately explains the data. That is, the RMSD was used to detect items exhibiting unexpected response probabilities across different levels of the latent variable. However, the RMSD is also widely used in other contexts. For example, it can be used for measurement invariance testing or differential item functioning (DIF) analysis (e.g., Buchholz & Hartig, 2019; Joo et al., 2021, 2024; Tijmstra et al., 2020). The reference values derived in this study are not generalizable to these contexts.

### Closing Remark

This study provides two rigorous yet accessible approaches to evaluating item fit using a pseudocount-based RMSD: (1) statistical significance testing using the PP-PPMC where replicated data are drawn from the posterior predictive distribution and (2) empirical derivation of reference values where replicated data are drawn from the predictive distribution. Under a sufficiently large number of examinees (e.g., 1,000 or more) and items (e.g., 40 or more), the two approaches demonstrated comparable power (true-positive rate) and Type I error rate (false-positive rate). Under data-limited scenarios, such as smaller sample size and/or shorter test length, the cutoff threshold approach outperformed the PP-PPMC method, yielding a significant increase in the true-positive rates with only a small to moderate increase in the false-positive rates. The cutoff threshold approach also performed well under high levels of data missingness common in a large-scale assessment context. However, in cases where researchers cannot afford a high false-positive rate, the PP-PPMC approach offers a more conservative alternative that strictly controls the Type I error rate, even in data-limited scenarios. These tools effectively complement the existing visualization techniques by enhancing the efficiency and accuracy of item fit evaluation. While the findings demonstrate a strong performance of these methods within the scope of the simulation studies, caution should be exercised when applying them beyond the simulated conditions.

## Supplemental Material

sj-pdf-1-epm-10.1177_00131644251369532 – Supplemental material for Evaluation of Item Fit With Output From the EM Algorithm: RMSD Index Based on Posterior Expectations Supplemental material, sj-pdf-1-epm-10.1177_00131644251369532 for Evaluation of Item Fit With Output From the EM Algorithm: RMSD Index Based on Posterior Expectations by Yun-Kyung Kim, Li Cai and YoungKoung Kim in Educational and Psychological Measurement

## Notes

1. For instance, in flexMIRT^®^ (Cai, 2024), users can extract the E-table with the commands ‘SaveEtbl = Yes’ and ‘SaveDBG = Yes’ (Houts & Cai, 2024). In the TAM (Test Analysis Modules) package in R, the RMSD can be obtained using the ‘IRT.itemfit’ function (Robitzsch et al., 2024).
