# Supplementary material for: Evaluation of Item Fit With Output From the EM Algorithm: RMSD Index Based on Posterior Expectations
Source: Educ Psychol Meas. 2025 Oct 4;86(1):156–90. doi: 10.1177/00131644251369532 (PMC12496452; doi:10.1177/00131644251369532)
Supplement: Supplementary file 1 [file sj-pdf-1-epm-10.1177_00131644251369532.pdf]

Supplementary Materials

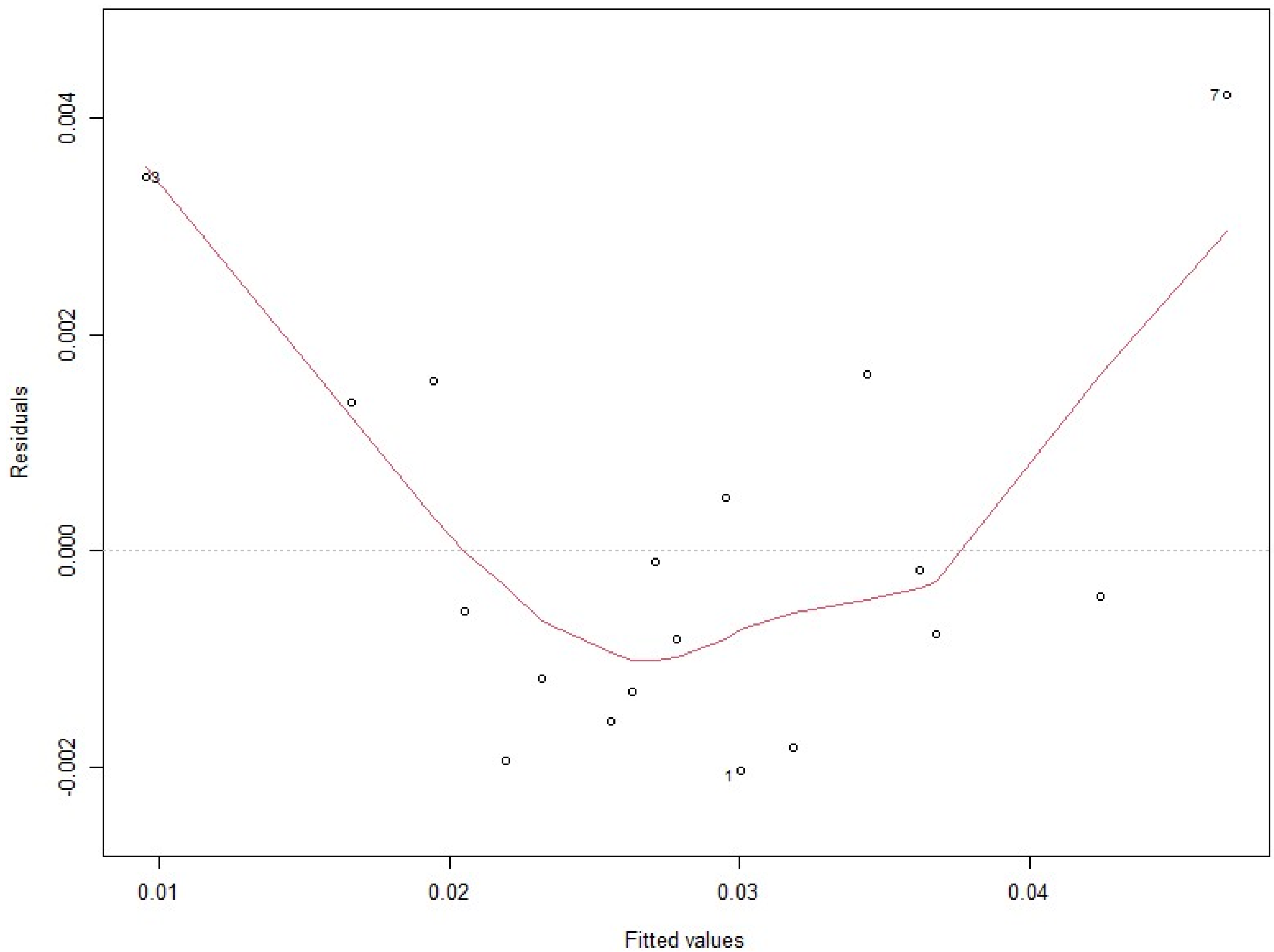

(a) Residuals vs Fitted

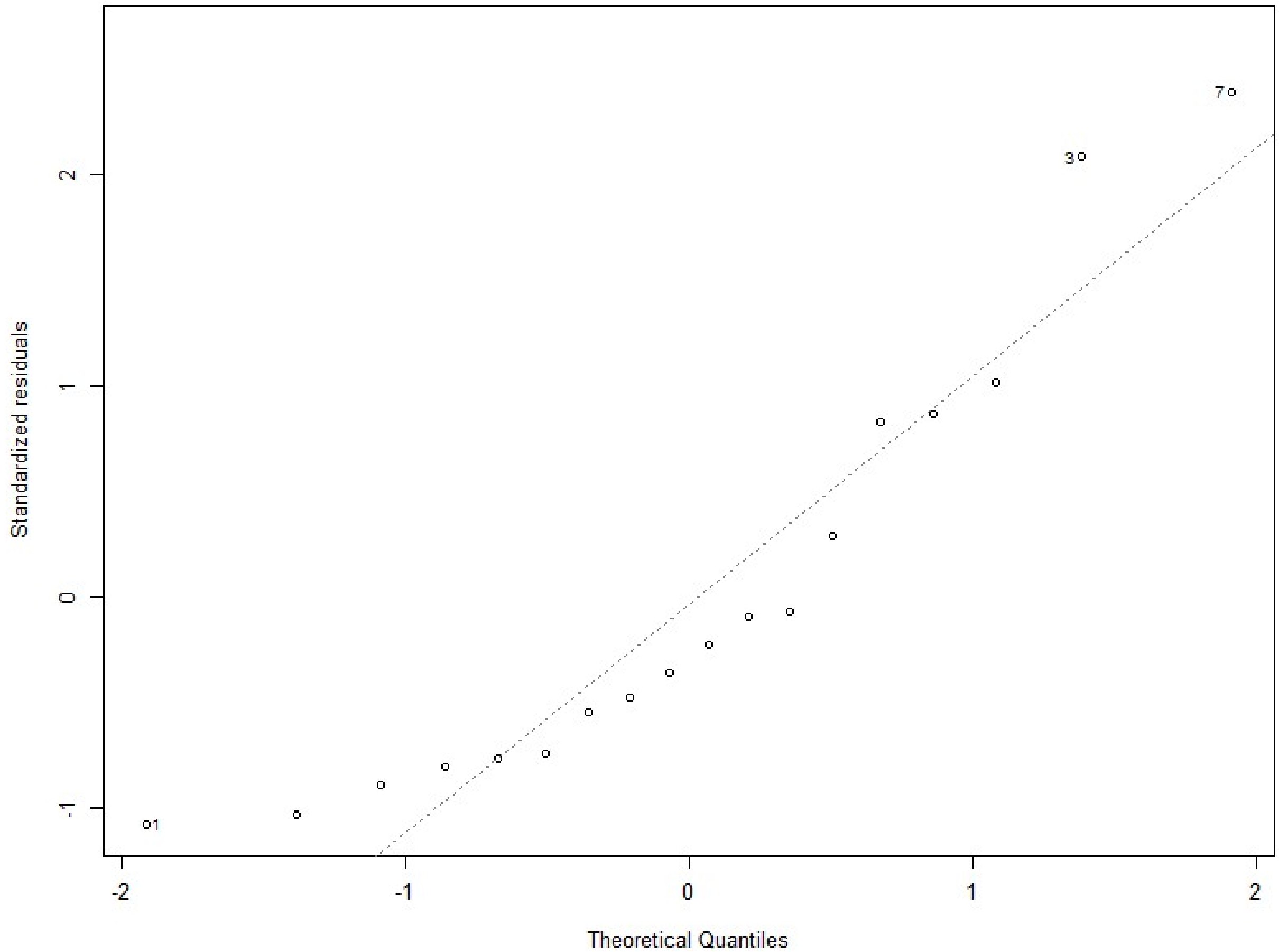

(b) Q-Q Residuals

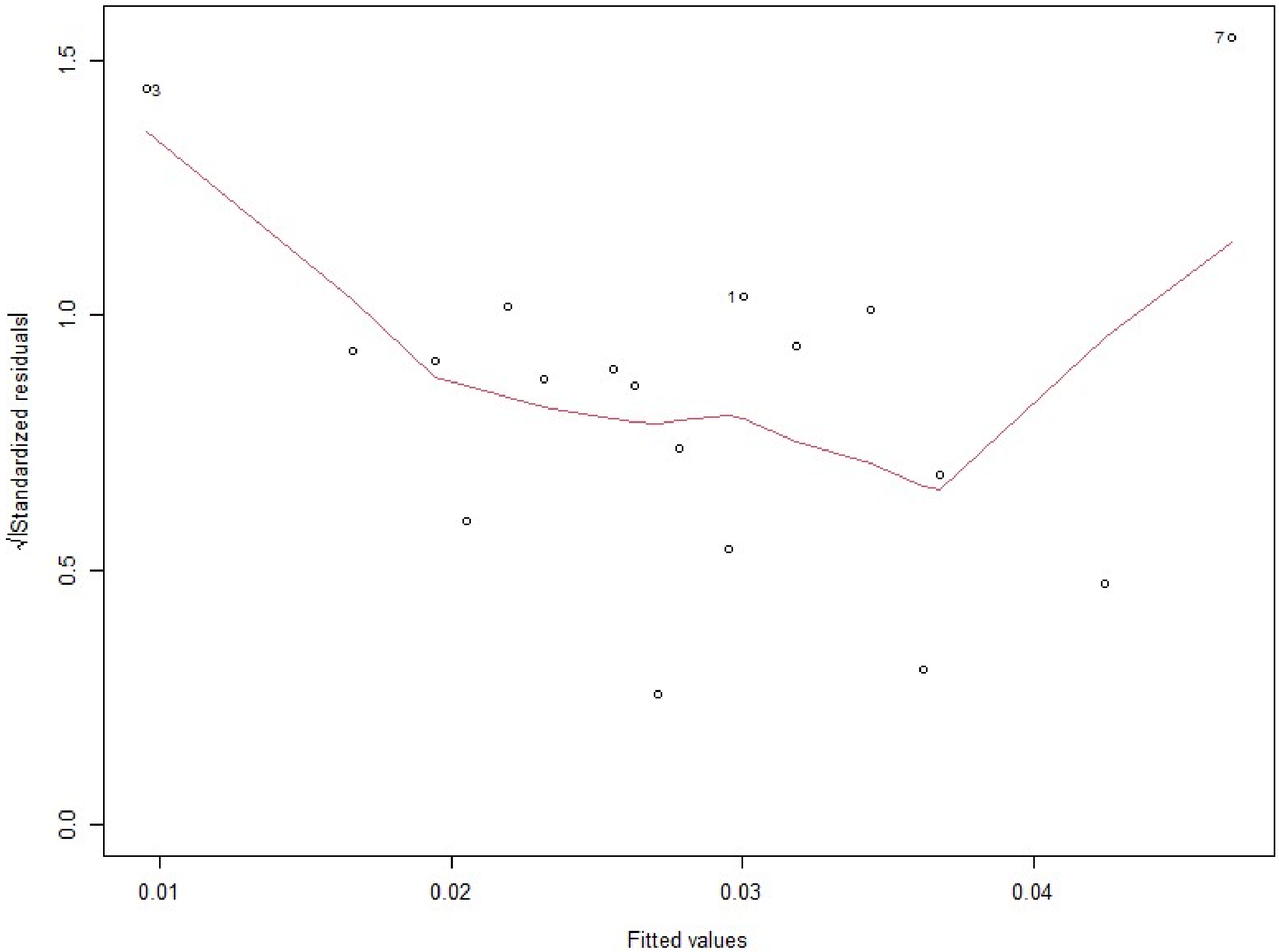

(c) Scale-Location

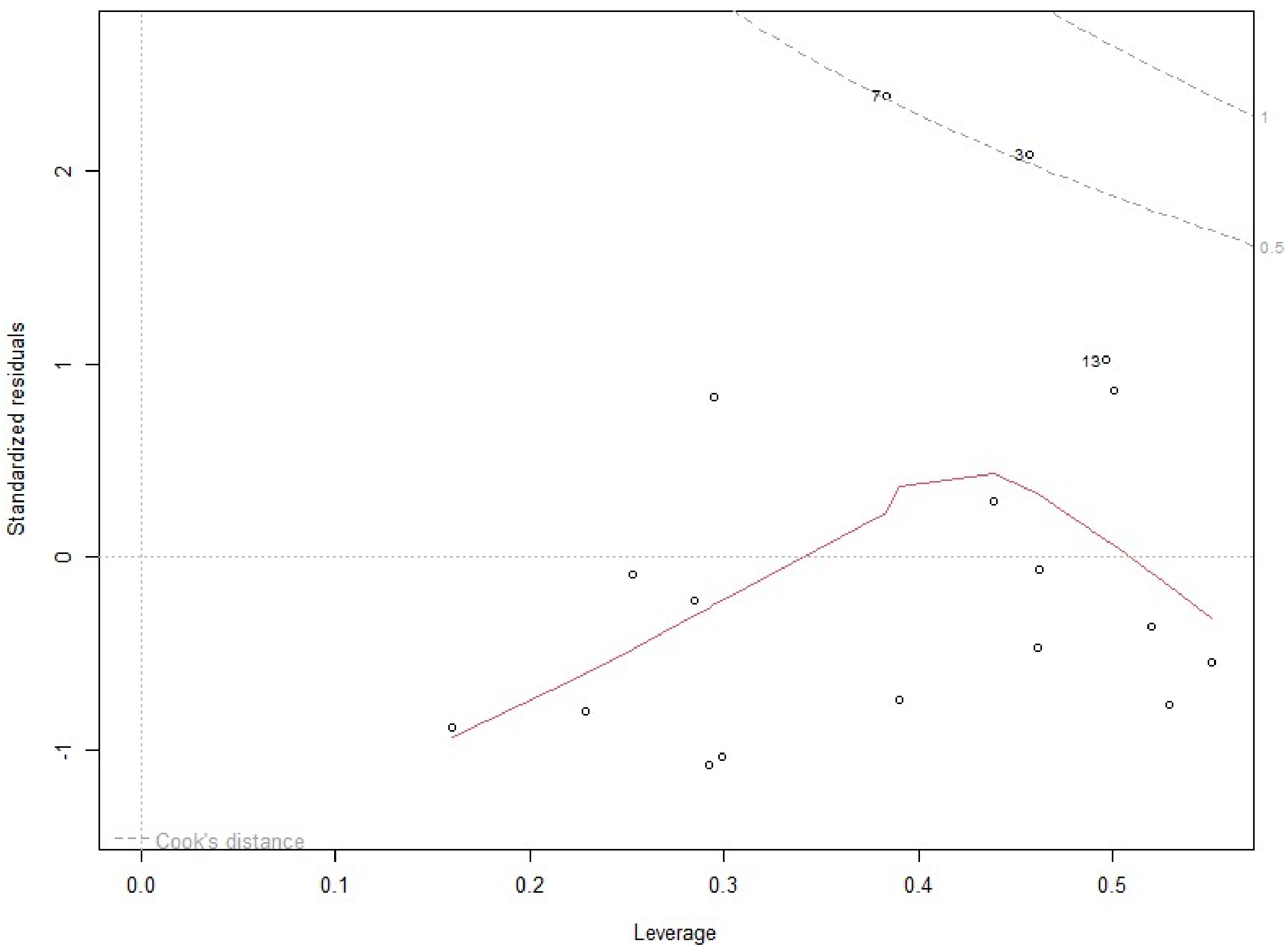

(d) Residuals vs. Leverage
