# Supplementary material for: Evaluation of Item Fit With Output From the EM Algorithm: RMSD Index Based on Posterior Expectations
Source: Educ Psychol Meas. 2025 Oct 4:00131644251369532. Online ahead of print. doi: 10.1177/00131644251369532 (PMC12496452; doi:10.1177/00131644251369532)
Supplement: sj-pdf-1-epm-10.1177_00131644251369532 – Supplemental material for Evaluation of Item Fit With Output From the EM Algorithm: RMSD Index Based on Posterior Expectations [file sj-pdf-1-epm-10.1177_00131644251369532.pdf]

Supplementary Materials

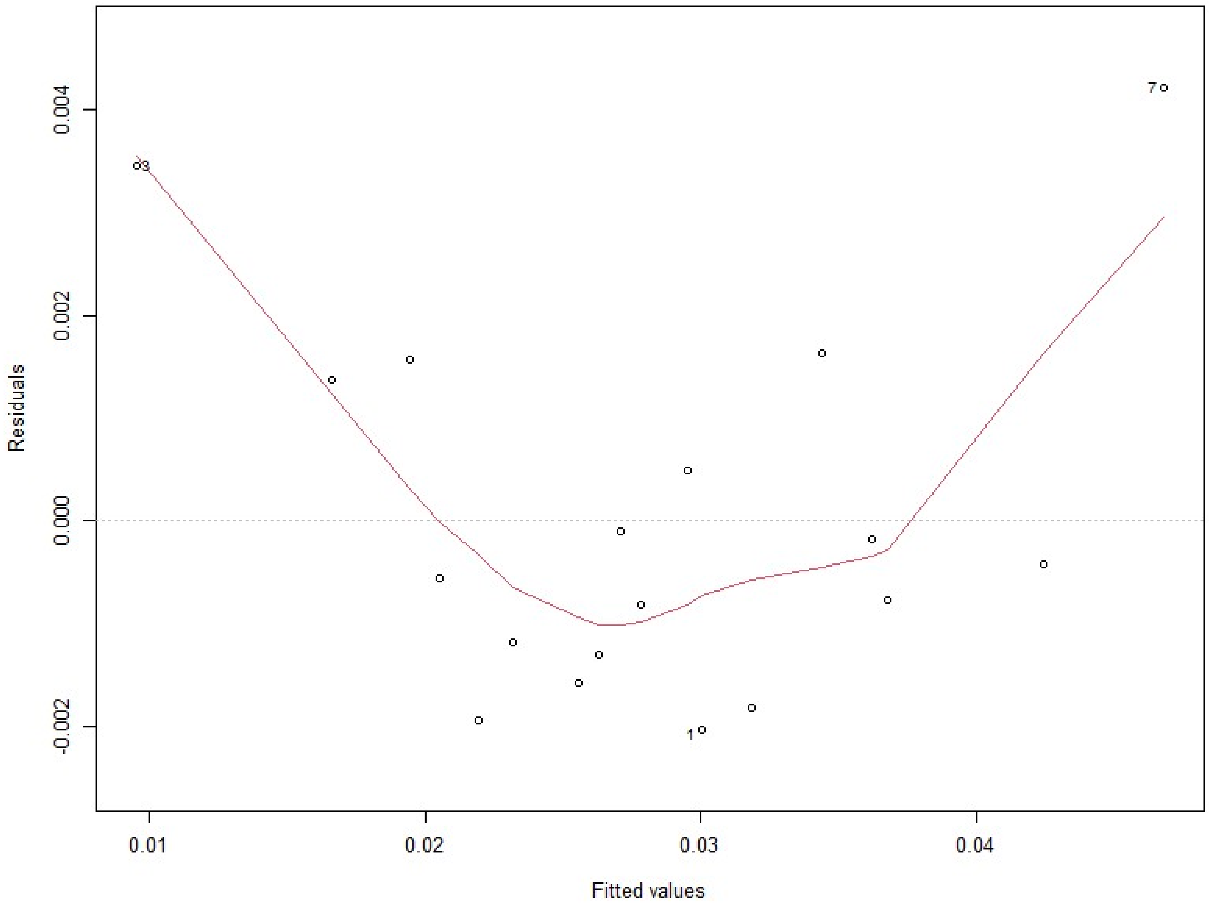

(a) Residuals vs Fitted

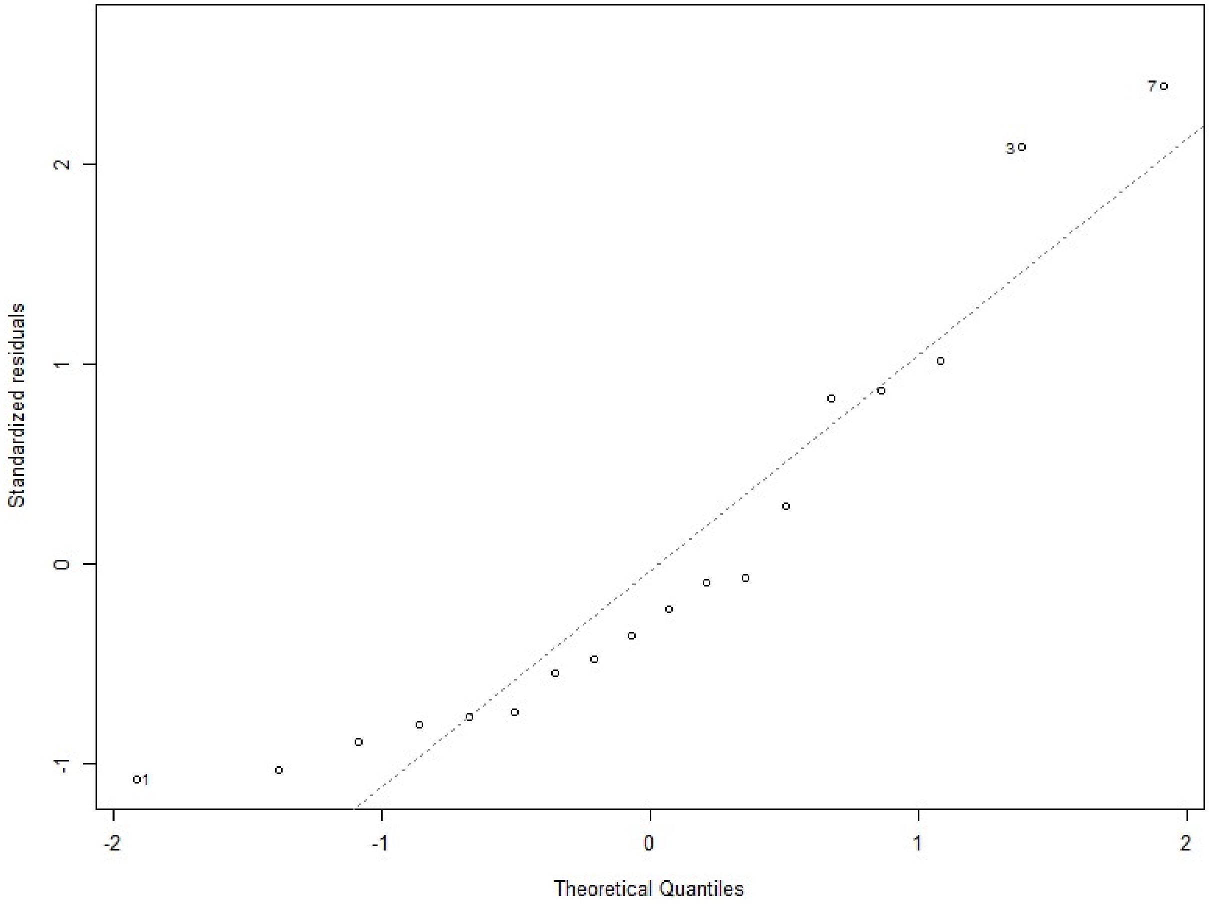

(b) Q-Q Residuals

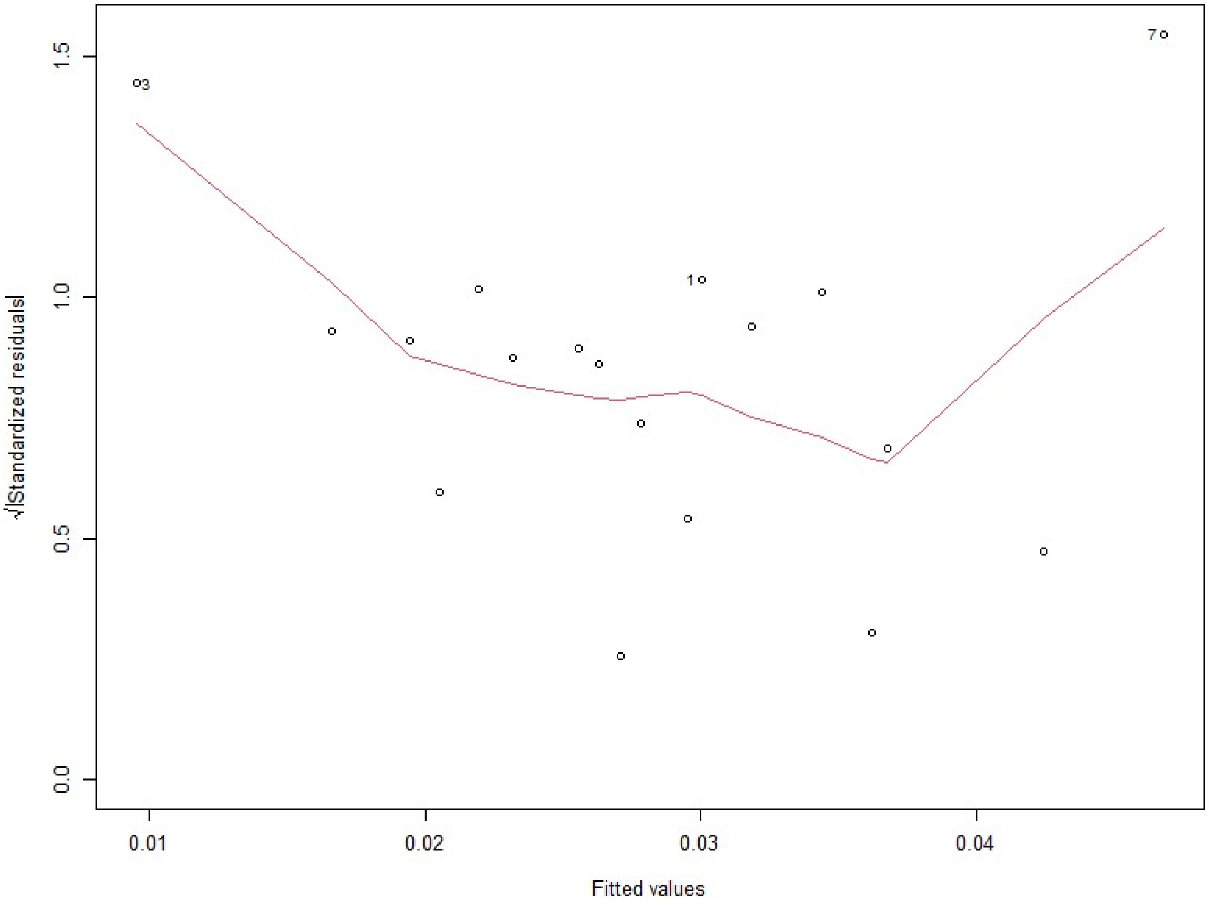

(c) Scale-Location

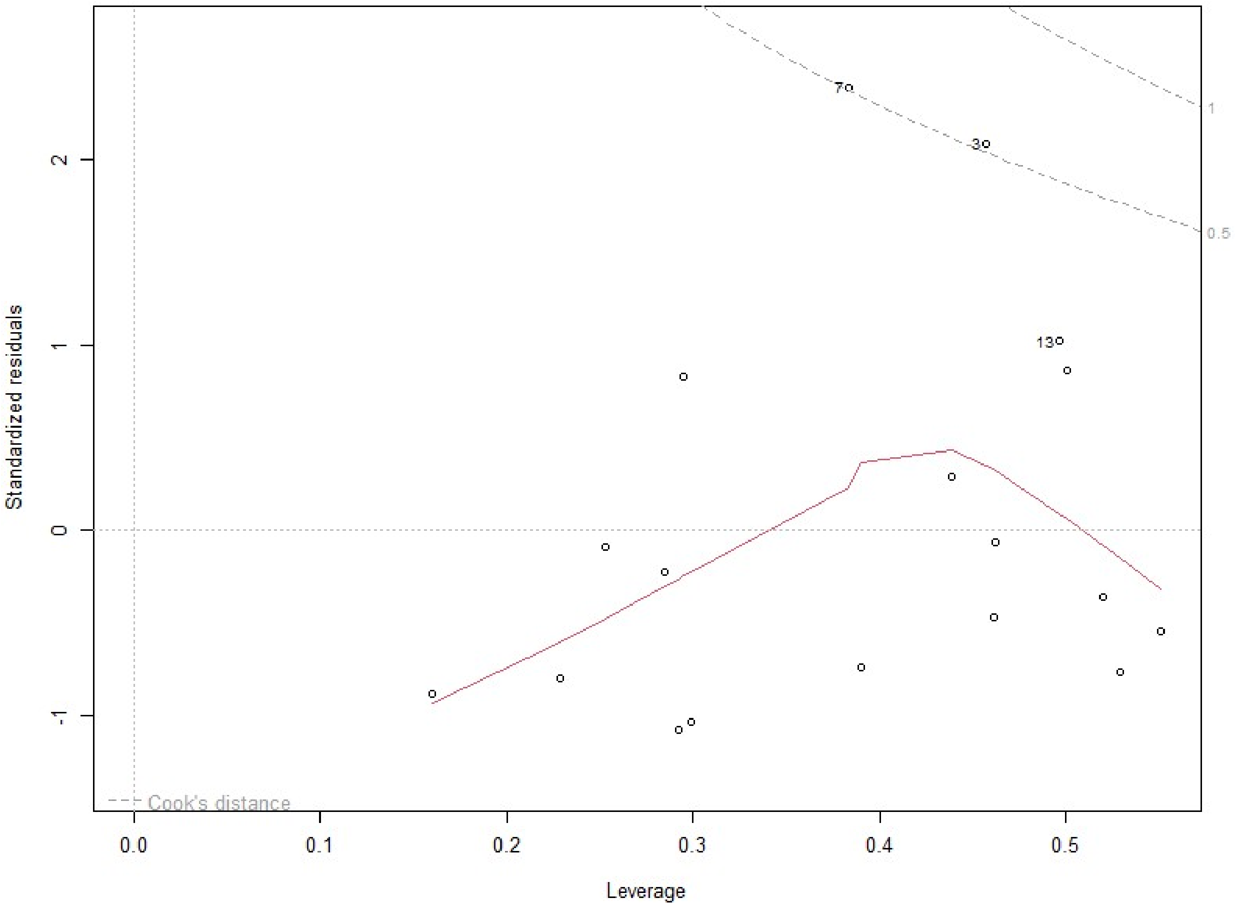

(d) Residuals vs. Leverage
